# Detection of Bacterial Internalization in Lettuce (*Lactuca sativa*) Leaves Grown in Aquaponic Systems with Nile Tilapia (*Oreochromis niloticus*) Under Microbial Challenge

**DOI:** 10.3390/biology15070559

**Published:** 2026-03-31

**Authors:** Angélica Adiação Jossefa, Leonildo dos Anjo Viagem, Karoline Moreira Barbuio, Brunno da Silva Cerozi, Sebastian Wilson Chenyambuga

**Affiliations:** 1 Department of Animal, Aquaculture and Range Sciences, Sokoine University of Agriculture, Morogoro P.O. Box 3004, Tanzania; leonildo.viagem@sacids.org (L.d.A.V.); chenya@sua.ac.tz (S.W.C.); 2 SACIDS Africa Center of Excellence for Infectious Diseases, SACIDS Foundation for One Health, Sokoine University of Agriculture, Morogoro P.O. Box 3004, Tanzania; 3 Department of Animal Science, College of Agriculture, University of São Paulo, Avenue Padua Dias, 11, P.O. Box 9, Piracicaba 13418-900, SP, Brazil; karolinembarbuio@usp.br (K.M.B.); brunno.cerozi@usp.br (B.d.S.C.); 4 Department of Agrarian Production, Higher School of Rural Development, Eduardo Mondlane University, Maputo 1100, Mozambique; 5 Institute of Natural Resources and Environment, Rovuma University, Cabo Delgado 3200, Mozambique

**Keywords:** biochemistry assays, challenge, contamination, human pathogens, lettuce leaves

## Abstract

Aquaponic systems are increasingly recognized as sustainable technologies for integrated fish and vegetable production. However, concerns remain regarding the potential internalization of human pathogens into vegetables grown in these systems. This study assessed the risk of pathogen internalization in lettuce leaves grown in aquaponic systems with Nile tilapia challenged with *Escherichia coli* or *Vibrio cholerae*. The system comprised nine fish tanks, eighteen hydroponic pipes, and eighty-one lettuce plants, with tanks assigned to three treatments. Samples of water, fish gut, fish blood, and lettuce leaves were collected. Microbiological analyses included selective culture, biochemical assays, and molecular identification. Although colonies consistent with *E. coli* and *V. cholerae* were recovered on selective media, molecular sequencing identified other bacterial species, including *Aeromonas sp*., *Aeromonas caviae*, *Aeromonas veronii*, *Enterobacter hormaechei*, and *Citrobacter freundii.* The findings indicate that conventional culture-based methods may produce false-positive results and highlight the importance of molecular confirmation. Notably, pathogenic bacteria associated with tilapia were detected and appeared capable of disseminating through the system and internalizing into lettuce tissues. This result highlights the need for biosecurity measures, contamination monitoring, and the combined use of conventional and molecular diagnostic tools to ensure accurate pathogen detection and compliance with international food safety standards.

## Simple Summary

Aquaponic systems are a sustainable way to produce fish and vegetables together while using less water and space. However, there are concerns that harmful bacteria from fish or water could enter the vegetables grown in these systems and pose risks to human health. This study investigated whether disease-causing bacteria could move from the fish and reach lettuce leaves through root uptake. The system included fish tanks connected to pipes where lettuce was grown. Some fish were exposed to two bacteria commonly linked to foodborne illnesses. The samples were collected from the water, fish blood, fish intestine, and lettuce leaves. They were analyzed using laboratory tests that detect and identify bacteria. Initial tests suggested the presence of the injected bacteria, but more precise genetic analyses showed that other types of bacteria were present instead. These bacteria were able to spread through the system and enter lettuce tissues. These findings show that commonly used laboratory methods may sometimes give misleading results and that more accurate identification methods are important. The study highlights the need for good hygiene practices and regular monitoring in aquaponic systems to ensure that the vegetables produced are safe for consumers.

## 1. Introduction

Aquaponic systems have emerged as an innovative and sustainable approach to food production, integrating aquaculture and hydroponics into a single, recirculating system. As global demand for both fish and vegetables continues to increase, driven by population growth and greater awareness of healthy diets, aquaponics offers a promising solution for the simultaneous production of animal protein and fresh produce. By combining fish rearing with soilless plant cultivation, aquaponics maximizes resource efficiency while reducing environmental impacts associated with conventional agriculture [1,2,3]. In aquaponic systems, fish are raised in tanks where their metabolic waste, primarily in the form of ammonia, accumulates in the water. Through microbial nitrification processes, ammonia is converted into nitrites and subsequently into nitrates, which serve as nutrients for plants. The plants, typically grown hydroponically, absorb these nutrients from the water, thereby purifying it before it is recirculated back to the fish tanks. This closed-loop design minimizes water exchange and reduces the need for synthetic fertilizers, as plant nutrients are derived directly from fish waste [4,5,6]. The growing interest in sustainable food systems has further increased attention to aquaponics as a viable strategy for helping to feed the expanding global population [7,8]. Its capacity to produce both fish and vegetables in limited spaces, while using significantly less water than soil-based agriculture, makes it particularly attractive in regions facing land and water constraints. Moreover, the absence of soil reduces exposure to soil-borne plant pathogens and eliminates the need for many conventional agrochemicals. Despite these advantages, aquaponic systems also raise important food safety concerns. Because plants are cultivated in water containing fish excreta, there is a potential risk of contamination with pathogenic microorganisms. The consumption of raw vegetables, especially leafy greens such as lettuce, has been frequently associated with foodborne illnesses linked to enteric pathogens [2,9,10,11]. Consequently, when vegetables are grown in aquaponic systems connected to fish production, microbiological safety must be carefully monitored and managed [12,13,14].

Aquaponics comprises multiple compartments, such as a fish tank, water column, hydroponic unit, biofilter, sump, solids tank, and fish. These compartments harbor highly diverse and functionally distinct microbial communities, reflecting the ecological complexity of these integrated systems. Collectively, these compartments host a wide range of bacterial phyla, such as Proteobacteria, Actinobacteria, Bacteroidetes, Firmicutes, Acidobacteria, Nitrospirae, Planctomycetes, and Fusobacteria. These phyla include microorganisms that play essential roles in nutrient cycling, organic matter degradation, and the maintenance of microbial balance within the system. Nevertheless, they also encompass potential human pathogens, which raises concerns in the context of food safety [15,16,17,18,19,20].

Nile tilapia is commonly used in aquaponic systems because of its resilience, tolerance to variable water quality conditions, and resistance to disease [11]. However, tilapia harbor a diverse microbiota that may include pathogenic bacteria for humans. Among these, *Escherichia coli* and *Vibrio cholerae* have been identified within tilapia-associated microbial communities [21,22,23,24,25,26]. These bacteria can proliferate in aquatic environments and, if ingested through contaminated food, colonize the human gastrointestinal tract, causing symptoms such as diarrhea, abdominal cramps, nausea, vomiting, and fever [27,28,29]. A major concern is the possibility that fish may serve as reservoirs of human pathogens.

Research conducted in hydroponic, soil-based, and some aquaponic systems has demonstrated that human pathogens can be internalized into plant tissues through root uptake, eventually reaching edible leaves [30,31,32,33,34]. However, relatively few studies have specifically examined the internalization of human pathogens in fully integrated aquaponic systems. The recirculating nature of these systems may facilitate the persistence and distribution of microorganisms across different compartments, including fish tanks, biofilters, and plant beds [35]. Circulating water can act as a transmission pathway, potentially transferring pathogens from fish to crops and thereby compromising vegetable safety and marketability [36]. These concerns highlight the need for rigorous scientific evaluation of pathogen dynamics in aquaponic environments. In particular, it is essential to determine whether fish can function as reservoirs of human pathogens and whether these microorganisms can disseminate through recirculating water and become internalized in plant tissues [25]. Therefore, this study aimed to evaluate the internalization of human pathogens in lettuce leaves grown in aquaponic systems with Nile tilapia under microbial challenge. The hypothesis of the study was that human pathogenic bacteria introduced into or residing in Nile tilapia would spread through recirculated water and be internalized into lettuce leaves via root absorption, thus representing a potential risk to food safety.

## 2. Materials and Methods

### 2.1. Experimental Design

**Figure 1:**
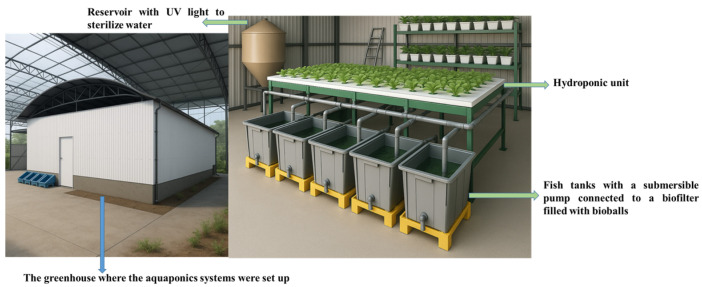
The greenhouse where the aquaponic system was set up, and the respective system used to raise Nile tilapia and grow lettuce.

The experiment design and aquaponic system setup were previously described by [37]. The study was conducted at the Fish Farming Center of the Department of Animal Science, Luiz de Queiroz College of Agriculture, University of São Paulo, Brazil, from March to May 2025. Briefly, the experiment followed a completely randomized design with three treatments and three replicates per treatment. T_1_ (control, i.e., fish not inoculated with bacteria), T_2_ (fish challenged with *Escherichia coli* and T_3_ (fish challenged with *Vibrio cholerae*). The aquaponic system consisted of 9 fish tanks, 18 hydroponic pipes, and 81 lettuce seedlings. Each fish tank was stocked with 16 Nile tilapia juveniles, and hydroponics pipers contained 9 plants (Figure 1). The fish tanks were equipped with an air stone and a submersible pump (Jeneca, IPF-5990-10V, Huanggang, China) connected to a biofilter. The pump pumped water from the fish tanks to the hydroponic units, with water returning by gravity to the fish tanks. The hydroponic units were illuminated with LED lamps (ES-600Q-72A, SawGlant, Shanghai, China) under an 18:6 h light–dark photoperiod.

Before the fish were introduced, the aquaponic system was thoroughly cleaned with soap and water, rinsed with bleach, and subsequently disinfected by filling it with water containing 240 ppm of hydrogen peroxide for one hour. After disinfection, the hydrogen peroxide solution was drained, and the system was rinsed to remove any residual disinfectant. To verify the effectiveness of this disinfection process, sterile distilled water was added to the system and maintained for 24 h. Subsequently, 500 mL water samples were collected from each tank and inoculated onto Eosin Methylene Blue (EMB) agar for *E. coli*, Thiosulfate–Citrate–Bile Salts–Sucrose (TCBS) agar for *V. cholerae*, and Tryptic Soy Agar (TSA), a non-selective medium. In addition, surface swabs were collected from different parts of the system and cultured on the same media. After confirming the absence of bacterial growth, the fish tanks were filled with deionized water purified through reverse osmosis (BioThec, CM-230, Piracicaba, Brazil) and subsequently sterilized using a UV filter (OceanTech Professional Aquarium, UV-567, 8000 L/min, Piracicaba, Brazil). The treated water was also tested and confirmed to be free of bacterial contamination on EMB agar, TCBS agar, and TSA.

### 2.2. Lettuce Seedling Establishment

Pellet seeds of *Lactuca sativa* were germinated in a hydroponics tray disinfected by immersion in water containing 240 ppm of hydrogen peroxide for 24 h. The trays were filled with a plant growth substrate (cod 75, rice straw, Carolina Soil, Piracicaba, Brazil) that had been sterilized in an autoclave (AV-75, Phoenix, Araraquara, Brazil) at 121 °C for 15 min. The seedlings were irrigated twice daily with water from another aquaponic system that had been previously established and also sterilized in the same autoclave under the same conditions. The seedlings were grown in a greenhouse under natural photoperiod conditions. After 28 days, they were tested for bacterial contamination and subsequently transplanted into the aquaponic system.

### 2.3. Stocking of Nile Tilapia Juveniles

Nile tilapia juveniles (average weight of 30.7 ± 0.03 g) were obtained from the Department of Animal Science, Luiz de Queiroz College of Agriculture, University of São Paulo, Brazil. The fish were quarantined for 15 days in two tanks, each with a capacity of 1000 L, with continuous well water exchange. After this period, water exchange was interrupted, and hydrogen peroxide (30 ppm) was added to each tank for 1 h to disinfect the fish. The fish were fed *ad libitum* twice daily at 9:00 a.m. and 5:00 p.m. The feed was manufactured at the fish farming center and formulated to contain 36% crude protein and 300 kcal of metabolizable energy per kilogram, and was stored at 4 °C until use. The fish were introduced into the aquaponic system 15 days before lettuce seedlings were added. The microbiology characterization of these fish has been previously reported by [37].

### 2.4. Challenging Nile tilapia

The source and concentration of *Escherichia coli* ATCC 8739 and *Vibrio cholerae* ATCC 9458 are described by [37]. The bacterial inoculum was cultured in selective media: Eosin Methylene Blue (EMB) agar for *E. coli* and Thiosulfate–Citrate–Bile Salts–Sucrose (TCBS) for *V. cholerae*. Colony-forming units (CFUs) were estimated using a spectrophotometer. Twenty days after the introduction of lettuce seedlings into the aquaponic system, samples of fish (blood and feces), water, and lettuce leaves were analyzed for the presence of *E. coli* and *V. cholerae*. Detection was performed according to the methodologies described by [38,39], which included plate culture, biochemistry assays, DNA extraction, PCR, and sequencing. Subsequently, fish in T_2_ and T_3_ were experimentally challenged. Fish in T_2_ were injected with 0.5 mL of 10^4^ CFU/mL of *Escherichia coli*, while fish in T_3_ received 0.5 mL of 10^4^ CFU/mL of *Vibrio cholerae*. The inoculum concentration (10^4^ CFU/mL) was selected based on previous experimental infection studies demonstrating sufficient bacterial exposure to evaluate colonization while minimizing acute mortality [40]. The bacterial strain suspension was administered via intraperitoneal injection using sterile hypodermic needles.

### 2.5. Water Quality Parameters

The water quality parameters in aquaponic systems were measured as described by [31]. Temperature and dissolved oxygen (DO) were measured at 8:00 a.m. and 4:00 p.m. using an oximeter (AT160, Alfakit, Florianopolis, SC, Brazil). pH was recorded at 4:00 p.m. using a pH-meter (SL110, SOLAR instrumentation Ltd., São Paulo, Brazil), and electrical conductivity (EC) was measured weekly using a conductivity meter (mCA-150, MS TecnoPon Special Equipment Ltd., Piracicaba, Brazil). Ammonia and nitrite levels were also measured weekly using LabConTest, Piracicaba, Brazil and ProDac chemical kits, Piracicaba, Brazil, respectively.

### 2.6. Sample Collection and Microbiological Analysis

Microbial analyses were conducted at the Fish Microbiology Laboratory, Department of Food Science and Technology, College of Agriculture, Luiz de Queiroz, University of São Paulo, Piracicaba, Brazil. Samples were collected from the fish gut, fish blood, water, and lettuce leaves 20 days after the introduction of lettuce seedlings into the systems and five days after Nile tilapia were challenged with pathogenic bacteria. Sample collection and processing followed the methodologies described by [38,39]. These procedures included culturing samples on EMB agar for the isolation of *E. coli* and TCBS agar for the isolation of *V. cholerae*. Subsequently, the presumptive colonies were subjected to biochemistry tests: triple sugar iron (TSI) agar for *E. coli* and the oxidase test for *V. cholerae*. Colonies that tested positive in the biochemistry tests were then used for DNA extraction, Polymerase Chain Reaction (PCR), and sequencing.

For water sampling, 500 mL of water was collected from each tank in the morning. From this volume, 50 mL was filtered through a 0.45 µm syringe membrane filter. The membrane was then rinsed with 2 mL of buffered peptone water (BPW). For Nile tilapia sampling, three fish per tank were randomly selected after 24 h of fasting. For blood collection, the fish were anesthetized on ice, and the skin surface was disinfected with sterile gauze soaked in 70% ethanol. Blood samples were drawn from the caudal vein using sterile hypodermic needles (SR-Products LA Salud SA, Pedro Juan Cabellero, Paraguay) containing 1% EDTA to prevent clotting. The collected blood was transferred into sterile 2 mL centrifuge tubes, which were placed on a polypropylene rack kept on ice. After blood collection, the same fish were euthanized using benzocaine (1 g/10 L). The intestines were aseptically removed for fecal sampling. To remove benzocaine residues, the intestines were rinsed with phosphate-buffered saline (PBS). Feces were obtained by squeezing the intestine into a sterile 2 mL centrifuge tube, which was also kept on ice. Subsequently, 0.2 mL aliquots of both blood and fecal samples were transferred into tubes containing 2 mL of buffered peptone water.

For lettuce sampling, the number of plants harvested from the tanks and the sample weight used for bacterial detection were determined according to [12]. However, a modification was made to the sample preparation step. Instead of homogenizing 25 g of lettuce in 225 mL of buffered peptone water, as described in their protocol, this study used 10 g of lettuce leaves homogenized in 100 mL of buffered peptone water. Three heads of lettuce were randomly collected from each tank. The leaves were surface-disinfected by immersion in 0.3% hydrogen peroxide for 1 min, followed by 70% ethanol for 10 s, and finally rinsed with sterile water to remove disinfectant residues. To evaluate the effectiveness of the disinfection procedure, swab samples were collected from six randomly selected lettuce leaves, while two untreated leaves (without disinfection) served as controls. The swabs were cultured on EMB agar for *E. coli*, TCBS agar for *V. cholerae*, and TSA (a non-selective medium). After disinfection, 10 g of leaves were aseptically collected using sterile scissors, transferred into sterile bags containing 100 mL of buffered peptone water, and crushed and homogenized for 2 min using a stomacher (EQ-061, ITR, São Paulo, Brazil).

After the addition of buffered peptone water, all samples were homogenized using a vortex machine (MX-E, NOVA INSTRUMENTS, São Paulo, Brazil) and incubated for 6 h. Subsequently, 100 µL of each sample was plated on EMB and TCBS agar using sterile glass Drigalski loops. The plates were incubated at 37 °C for 24 h. After incubation, colonies displaying a black metallic sheen on EMB and yellow colonies on TCBS were considered presumptive *E. coli* and *V. cholerae*, respectively, and were subjected to biochemical confirmation. Presumptive *E. coli* colonies were cultured on Triple Sugar Iron (TSI) agar for 24 h, while presumptive *V. cholerae* colonies were tested using the oxidase test. Biochemistry assays were performed only on colonies obtained from plate culture. All bacterial analyses were carried out in a biological safety cabinet (SBIIB21584/p, FIL TERFLUX Laboratory Equipment Ltda, São Paulo, Brazil), and all materials used were sterilized in an autoclave (CS, PRISMATEC Industry and Commerce Ltda, Catanduva, São Paulo, Brazil).

### 2.7. DNA Extraction from the Colonies

After confirmation by biochemical tests, presumptive colonies of each bacterium were inoculated into Luria–Bertani (LB) Broth agar and incubated for 24 h. Subsequently, 2 mL of each culture was centrifuged at 10,000 rpm for 5 min at 25 °C. The supernatant was discarded, and the pellet was washed with 1 mL of PBS, vortexed, and centrifuged again under the same conditions. After the washing step, the pellets were suspended in 1 mL of PBS and heated in a water bath at 95 °C for 10 min for cell lysis. The tubes were then centrifuged again under the same conditions, using a HITACHI-CF16RN centrifuge (Hitachi Ltd., Hitachi, Ibaraki, Japan). The DNA extracted through this procedure was sent to the LABMIC Biomol e Micotoxinas laboratory, Department of Food Science and Technology, University of São Paulo, Brazil. The DNA served as a template for PCR to detect the target bacteria through amplification of species-specific genes (*mdh* and *rfbE* genes of *E. coli*, and *ompW* and *ctxA* genes of *V. cholerae*).

### 2.8. PCR Amplification

For the amplification of specific genes from presumptive *E. coli* and *V. cholerae* colonies, the genes and corresponding primers listed in Table 1 were used. In parallel, the 16S rRNA gene (V3-V4 regions) was also amplified. For this purpose, the universal oligonucleotide primers 27F (5′-AGAGTTTGATCMTGGCTCAG-3′) and 533R (5′-TTACCGCGGCKGCTGGCACG-3′) were employed. PCRs were performed using PCRBIO Taq Mix 2X (PCR Biosystems, New York, NY, USA) in a final volume of 25 µL, containing 12.5 µL of the master mix, 0.3 µM of each primer, and 10–50 ng of template DNA, and ultrapure water to adjust the final volume. The thermocycling conditions consisted of an initial denaturation at 95 °C for 3 min, followed by 35 cycles of denaturation at 95 °C for 30 s, annealing at 60 °C for 30 s, and extension at 72 °C for 30–60 s. A final extension step was performed at 72 °C for 5 min. The PCR products were visualized by electrophoresis on a 1.5% agarose gel stained with GelRed^®^.

### 2.9. PCR Product Purification

The amplified products were purified using the EasyPure^®^ PCR Purification Kit (TransGen Biotech, Beijing, China), following the manufacturer’s instructions. Briefly, five volumes of binding buffer were added to one volume of the PCR product, and the mixture was applied to the silica column provided by the kit. After centrifugation, the column was washed with 650 µL of the washing buffer previously supplemented with ethanol. The DNA was then eluted with 30 µL of nuclease-free water and recovered by centrifugation.

### 2.10. Sequencing

The purified amplicons were subjected to the Sanger sequencing reaction. This method is based on chain termination using fluorescent dideoxynucleotides (ddNTPs). The sequencing reaction was performed using the BigDye Terminator kit v 3.1, Thermo Fisher Scientific, Waltham, MA, USA, according to the manufacturer’s protocol. The reaction mixture included the sequencing primer (the same as the primers used for amplification), DNA polymerase, dNTPs, and labeled ddNTPs. The sequencing products were purified using purification columns to remove excess ddNTPs and subsequently separated by size using an automated genetic analyzer (ABI 3500xL Capillary Sequencer, Applied Biosystems, Foster City, CA, USA).

### 2.11. Sequencing Data Analysis

The consensus 16S rRNA gene sequence generated by Sanger sequencing was edited, trimmed, and exported in FASTA format. The sequence was compared with reference sequences using the Nucleotide BLAST (blastn) algorithm implemented in the BLAST v2.17.0 program at the National Center for Biotechnology Information (NCBI). Searches were performed against the “16S ribosomal RNA sequences (Bacteria and Archaea)” database using the “Highly similar sequences (megablast)” option with default parameters. Taxonomic identification was determined based on the highest-scoring hits, considering percentage identity, query coverage, E-value, and alignment score. An identity value ≥97% with high query coverage (≥95%) and an E-value approaching 0.0 was considered indicative of species-level identification, whereas lower similarity values were interpreted as genus-level affiliation or potential taxonomic novelty.

### 2.12. Statistical Analysis

Before statistical analysis, the distribution of water quality parameters was assessed for normality using the Shapiro–Wilk test. Homogeneity of variances among treatment groups was evaluated using Bartlett’s test. When the assumptions of normality and homoscedasticity were met, differences among treatments were analyzed using one-way analysis of variance (ANOVA). The statistical significance of treatment effects was determined using the F-test at a significance level of *p* < 0.05. Microbiological results were recorded as the presence or absence of presumptive bacterial colonies before and after challenge in each sample. To evaluate differences in contamination frequency across treatments, contingency table analyses (File S1 in the Supplementary Materials) were performed using Fisher’s exact test, given the small sample size. Statistical significance was considered at *p* < 0.05. For the *E. coli* samples in water before the challenge, as well as for *E. coli* in both feces and water after the challenge, statistical tests were not applied because all samples yielded the same result (presence of *E. coli* in all tanks).

## 3. Results

### 3.1. Water Quality Parameters

The results related to water quality parameters were previously published in [31] and are therefore summarized here. The temperature and nitrate remained stable among the treatments with an average of 25.83 ± 2.86 °C and 91.7 ± 14.46 mg/L, respectively. The mean dissolved oxygen (DO) concentration was 6.55 ± 1.01 mg/L. Electrical conductivity (EC) averaged 170.94 ± 39.79 μS/cm, while ammonia and nitrite concentrations were similar across treatments, averaging about 0.38 mg/L. Statistical analysis indicates that there were no significant differences in water quality parameters across treatments (*p* > 0.05).

### 3.2. Microbiological Analysis

The disinfection protocol used for the lettuce leaves, consisting of immersion in 0.3% hydrogen peroxide for 60 s followed by 70% ethanol for 10 s, and subsequently rinsing with sterile water, was effective in eliminating external bacteria. All leaf swab samples collected from leaves treated with this method showed no bacterial growth on EMB, TCBS, and TSA culture media, as shown in Figure S1 in the Supplementary Materials. Additionally, water samples collected from the system before the start of cultivation, as well as swabs taken from the system surface, also tested negative on the same culture media, confirming the absence of detectable bacterial contamination at baseline. For statistical analysis, Fisher’s exact test showed no significant differences in the frequency of presumptive bacterial detections between the control and pathogen-challenged treatments across the analyzed sample matrices (water, feces, and lettuce leaves) (*p* > 0.05).

### 3.3. Bacterial Contamination and Internalization Before the Challenge

#### 3.3.1. Plate Culture

To evaluate the contamination and internalization by *E. coli* and *V. cholerae* before the challenge, samples of water, fish blood, fish feces, and lettuce leaves were collected from each tank 20 days after the introduction of lettuce into the system. The growth of presumptive colonies of both bacteria was observed, as shown in Figure S2 in the Supplementary Materials. Water samples from all treatments tested positive for presumptive *E. coli*, while presumptive *V. cholerae* colonies were detected with increasing frequency from treatment 1 to treatment 3 (Table 2). No bacterial growth was observed in blood samples for either bacterium across all treatments (Table 3). Fecal samples were positive for presumptive *E. coli* in all treatments, and presumptive *V. cholerae* growth was also observed (Table 4). Lettuce samples showed presumptive contamination with both bacteria, depending on the treatment (Table 5).

#### 3.3.2. Biochemistry Tests and Sequencing Results

Before performing the microbial challenge on Nile tilapia, both positive and negative results were observed on the biochemistry tests for the two bacteria, as shown in Figure S3 in the Supplementary Materials. However, only a small number of fecal and lettuce samples tested positive in the biochemical analyses (Table 4 and Table 5) and were therefore submitted for sequencing. The sequencing results revealed that the target bacteria were not present in the analyzed samples, and the identified species are presented in Table 6.

### 3.4. Bacterial Contamination and Internalization After the Challenge

#### 3.4.1. Plate Culture

Samples of water, fish blood, fish feces, and lettuce leaves were collected five days after challenging Nile tilapia with *E. coli* and *V. cholerae*. In general, as presented in Table 2, Table 3, Table 4 and Table 5, treatment 1 showed water samples positive for presumptive colonies of *E. coli*, and the growth of presumptive colonies of *V. cholerae* was also observed, although in low numbers compared to *E. coli*. In treatments 2 and 3, in which the fish were challenged with *E. coli* and *V. cholerae*, respectively, the water samples showed presumptive growth of both bacteria. In the blood samples, bacterial growth was only observed in treatment 3, with growth of presumptive colonies of both bacteria. Regarding fecal samples, all treatments were positive for presumptive *E. coli* colonies, and the presumptive *V. cholerae* colonies were also observed more frequently in treatment 3. For lettuce leaves, presumptive colonies of both *E. coli* and *V. cholerae* were detected in all three treatments, with a higher occurrence of *V. cholerae* in treatment 3.

#### 3.4.2. Biochemistry Tests and Sequencing Results

The biochemical tests of both bacterial strains were positive in water, fish, and lettuce leaf samples (Table 2, Table 4 and Table 5). None of the blood samples were confirmed by biochemical tests (Table 3). After the challenge, the results were unchanged, and the injected bacteria were not detected in the samples. Sequencing analysis identified the species *Citrobacter freundii*, *Aeromonas caviae*, *Aeromonas veronii*, *Aeromonas* sp., *Enterobacter hormaechei*, and *Enterobacter mori*. These results are presented in Table 6 and in File S2 in the Supplementary Materials.

## 4. Discussion

### 4.1. Water Quality Parameters

The results revealed that all water quality parameters in the aquaponics system were within the range required to support optimal growth of both organisms. In this study, ammonia concentration decreased over time, while nitrate concentration increased, when the pH average was 7.12 ± 0.57. This pattern was probably due to the presence of nitrifying bacteria that converted ammonia into nitrate, making it available for plant nutrition. Previous studies have reported that in aquaponics, ammonia levels decrease while nitrate concentrations increase over time at an optimal pH ranging from 6.5 to 8.5 [43,44]. According to [44], the fluctuations in nitrogen compounds are driven by the development of nitrifying bacteria in the biofilter. The values for temperature, DO, nitrite, and EC were within the standard limits for aquaponic systems, indicating that the environmental temperature did not significantly influence water quality parameters in the systems. In the study by [43], dissolved oxygen ranged from 4.33 to 6.35 mg/L, while nitrite ranged from 0.045 to 0.089 mg/L in the aquaponics system used for the cultivation of tilapia and spinach. Studies by [6,45] reported that the optimal DO in aquaponics ranges between 4 and 8 mg/L, and EC may be 226.3 µS/cm.

### 4.2. Bacterial Contamination and Internalization Before and After the Challenge

The results obtained before and after the challenge indicated the absence of the target bacteria. However, the detection of potentially pathogenic bacteria in water and lettuce suggests that fish-associated microbiota may contribute to microbial dissemination within aquaponic systems. Nile tilapia naturally harbor microorganisms capable of contaminating water and becoming internalized in lettuce leaves. These observations suggest that fish may act as a reservoir and potential vectors for the dissemination of bacteria in aquaponic systems. Once introduced in the water, these microorganisms can colonize plant roots and subsequently internalize into the edible parts of lettuce, thereby creating a possible route for cross-contamination. The detection of these microorganisms in lettuce leaves supports the hypothesis of internalization via root uptake reported in early studies conducted in soil and hydroponic systems. Previous studies have shown that microorganisms from fish can persist in water and eventually reach the rhizosphere of the vegetables [46,47]. These microorganisms may be absorbed by plant roots and subsequently internalized into the edible plant tissues, including edible portions [31,33]. The internalization of microorganisms through root uptake has been observed in several crops, such as spinach, lettuce, and tomato [48,49]. Exposure of roots to contaminated water has been shown to result in the internalization of human pathogens in the aerial parts of plants [31]. These pathogens can enter production systems and become internalized into various plant tissues and organs by using the roots as an entry route [31,48,50]. Studies have also shown that pathogen internalization from water into plant tissues can occur as early as four hours after contact [51].

The absence of the target bacteria after the challenge indicates that these strains were unable to contaminate water or internalize into lettuce leaves. The failure of the challenge may be related to the host’s natural defense mechanisms, which could have limited or prevented colonization by these pathogens. Additionally, the challenge failure may be attributed to the presence of resident bacteria that could have competed with the introduced strains for space or nutrients, thereby inhibiting colonization. Another possible explanation is the concentration of the strains injected into Nile tilapia. This phenomenon has been reported in previous studies. For example, the finding of [52] revealed that the interactions between the fish’s resident microbiota and the injected bacteria resulted in the inhibition of pathogens. Furthermore, the co-infection of fish by pathogenic bacteria can be negatively impacted by the presence of commensal bacteria, which reduces the likelihood of pathogen survival [53,54]. These commensal bacteria can compete with the pathogens by occupying ecological niches and, consequently, protect the host from co-infection [55].

Pathogens injected via the intraperitoneal vein at concentrations of 0.1 × 10^6^ or 0.1 × 10^7^ were not detected in fish after 5, 12, and 27 days after the challenge. The DNA of these pathogens was also not detected by molecular methods [56]. In stagnant water systems, such as aquaponics, bacterial strains like *Vibrio cholerae* become undetectable approximately one week after inoculation. For instance, ref. [57] observed that the concentration of *V. cholerae* in stagnant water decreased from 10^7^ to 10^5^ CFU/mL in aquarium water and from 10^7^ to 10^4^ CFU/mL in the tilapia gut, eventually rendering the bacteria undetectable.

### 4.3. Sequencing Results

The study was developed with the hypothesis that human pathogens introduced into Nile tilapia could spread through the water circulating in the system and potentially reach the cultivated vegetables. However, molecular analyses did not confirm the presence of the injected pathogens in the fish, suggesting that these bacteria may not have persisted or colonized the host at detectable levels, which reduces the likelihood of their spread in the system and subsequent transfer to the vegetables. Thus, under the experimental conditions of this study, it was not possible to confirm the internalization of the inoculated pathogens. On the other hand, the results indicated that pathogenic bacteria naturally associated with fish can circulate in the system through recirculated water and potentially internalize into the vegetables through the roots. Cultivation in selective media, followed by biochemical tests, initially indicated the presence of *Escherichia coli* and *Vibrio cholerae*. However, molecular analyses and sequencing revealed that the isolated colonies did not correspond to the target bacteria, but rather to other bacterial species. This discrepancy can be explained by morphological similarities and phenotypic differences between different bacteria, which allow them to grow in selective media and generate incorrect presumptive identifications when molecular methods are not used for confirmation. According to [58], classical bacterial identification methods rely on the metabolic and phenotypic characteristics of each strain, which are often associated with the pathogen’s virulence factors. These biochemical techniques provide a reliable preliminary approach for identifying target bacteria based on their specific enzymatic activities, as also highlighted by [59]. Samples of water, animal, human, and environmental were plated on EMB agar and, after incubation at 37 °C for 24 h, colonies exhibited the typical metallic green sheen characteristics of *E. coli*, indicating rapid lactose fermentation. In addition, large yellow colonies characteristic of *V. cholerae* were detected on TCBS agar after 24 h of incubation. Both pathogens also showed positive reactions in TSI biochemical tests and oxidase assays, as well as the molecular analyses reported in the studies conducted by [38,39]. However, refs. [60,61] observed that the presumptive yellow colonies of *V. cholerae* isolated from plate culture were later identified by molecular techniques as *Aeromonas caviae* and *Aeromonas veronii*.

Although the target bacteria were not detected by the molecular method, the bacterial species identified in this study (*Aeromonas* sp., *Citrobacter freundii*, *Aromonas veronii*, *Aeromonas caviae*) are also pathogenic to humans. Species of the genus *Aeromonas* are commonly found in aquatic environments, including freshwater, and fresh fish and vegetables are recognized as potential reservoirs. A study conducted in Italy to investigate *Aeromonas* spp. contamination in vegetables reported that lettuce showed the highest contamination rates, with *A. caviae* and *A. veronii* being the most frequently detected species [62,63].

Lettuce produced in aquaponics systems is often consumed raw, and the presence of these pathogens represents a potential threat to food safety and public health, as they are associated with infectious diseases linked to the consumption of contaminated raw fish and vegetables. *Citrobacter freundii* is known to cause urinary, respiratory, and gastrointestinal infections in humans, with contaminated food identified as a major route of transmission [64,65]. Additionally, *A. caviae* and *A. veronii* are associated with gastrointestinal and bloodstream infections, and both have been isolated from fish, confirming their zoonotic potential [66,67]. Recently, a study reported the detection of *A. veronii* in young females following the consumption of contaminated fresh fish [68].

In this study, pathogen internalization was not detected in all lettuce samples. These findings highlight the complexity and variability of bacterial contamination and internalization processes in the aquaponic system, demonstrating that, even under controlled conditions, not all replicates exhibit the same colonization pattern. Previous studies have indicated that internalization through plant roots is rare and, when it occurs, it does not persist for more than seven days [32]. The translocation of human pathogens from the roots to leaves, followed by internalization, generally occurs to a limited extent [30,32,69,70]. Similarly, pathogens originating from animal manure introduced into the soil have been detected in only a small number of plants [71,72]. In the study by [51], only one-third of the analyzed samples tested positive for internalization. Among the different plant parts evaluated (roots, stems, and leaves), the highest pathogen concentration was observed in the roots, likely due to their direct contact with the contaminated water.

Previous studies have reported that one of the most important measures to ensure microbiological safety in aquaponic systems is rigorous water quality control, since water continuously circulates between fish tanks and vegetable growing units. This includes regular monitoring for microbiological contamination indicators, such as *Escherichia coli* [73]. In addition to monitoring, preventive measures should be adopted to avoid the introduction and spread of pathogenic microorganisms within the production system. These measures include water treatment, periodic sanitization of equipment, tanks, pipes, and work surfaces, as well as the implementation of good personal hygiene practices by system operators [74]. Another equally important strategy is ensuring proper feeding and sanitary management of fish, avoiding practices such as overfeeding, which can lead to the accumulation of organic matter and waste in the system and consequently favor the growth of pathogenic microorganisms [75]. Furthermore, several studies, such as [76,77], have demonstrated that probiotics can be effective in controlling pathogens in aquaculture systems. It can also be used in aquaponics to control pathogens in fish and, consequently, reduce bacterial internalization.

This study has limitations, including the absence of quantitative microbial counts, a lack of histological confirmation of tissue-level internalization, and evaluation at a single post-challenge time point. Future studies should incorporate quantitative PCR and multiple sampling intervals to better characterize bacterial dynamics.

## 5. Conclusions

The results showed that human pathogenic bacteria experimentally injected into Nile tilapia did not survive at detectable levels. Consequently, the hypothesis that bacteria injected into tilapia could survive and subsequently internalize into vegetables through the roots in aquaponic systems was not confirmed under the experimental conditions of this study. However, sequencing analyses revealed the presence of other human pathogens naturally associated with tilapia. This finding indicates that conventional identification methods may lead to false-positive results. Furthermore, the results suggest that bacteria naturally associated with Nile tilapia can spread within aquaponic systems and, under certain conditions, may internalize into lettuce tissues. While the experimentally introduced strains were not detected, the detection of opportunistic pathogens underscores the need for routine microbiological monitoring in aquaponic production. We recommend implementing food safety protocols to control human pathogens in fish before introducing lettuce into the aquaponics, which may improve the microbiological quality of the products. Additionally, combining conventional and molecular methods can improve the accuracy of microorganism identification.

## Figures and Tables

**Table 1:** Species-specific genes and corresponding primers used to target *E. coli* and *V. choerae* from the samples.

Gene	Target Organism	Primer	Size bp	Refence
*mdh*	*E. coli* (O157:H7)	F: 5′-GGTATGGATCGTTCCGACCT-3′	304	[39]
		R: 5′-GGCAGAATGGTAACACCAGAGT-3′		
*rfbE*	*E. coli* (O157:H7)	F: 5′-CAGGTGAAGGTGGAATGGTTGTC-3′	296	[41]
		R: 5′-TTAGAATTGAGACCATCCAATAAG-3′		
*OmpW*	*V. cholerae* O1	F: 5′-CACCAAGAAGGTGACTTTATTGTG-3′	588	[38]
		R: 5′-GAACTTATAACCACCCGCG-3′		
*ctxA*	*V. cholerae* O1	F: 5′-CTCAGACGGGATTTGTTAGGCACG-3′	301	[42]
		R: 5′-TCTATCTCTGTAGCCCCTATTACG-3′		

**Table 2:** Status of bacterial contamination in water samples before and after the challenge.

**Before the Challenge**													
**Sample**	**Bacteria Strain**	**T1 (Control)**				**T2 (Challenged with *E. coli*)**				**T3 (Challenged with *V. cholerae*)**			
		**Tank**	**Plate Culture**		**Biochemistry Test**	**Sample Number**	**Plate Culture**		**Biochemistry Test**	**Sample Number**	**Plate Culture**		**Biochemistry Test**
			**Plate 1**	**Plate 2**			**Plate 1**	**Plate 2**			**Plate 1**	**Plate 2**	
Water	*E. coli*	1	+	+	−	1	+	+	−	1	+	+	−
		2	+	+	−	2	+	+	−	2	+	+	−
		3	+	+	−	3	+	+	−	3	+	+	−
	*V. cholerae*	1	+	+	−	1	−	−	NT	1	−	+	−
		2	−	−	NT	2	+	+	−	2	+	−	−
		3	−	−	NT	3	+	+	−	3	+	−	−
**After the Challenge**													
**Sample**	**Bacteria Strain**	**T1 (Control)**				**T2 (Challenged with *E. coli*)**				**T3 (Challenged with *V. cholerae*)**			
		**Tank**	**Plate Culture**		**Biochemistry Test**	**Sample Number**	**Plate Culture**		**Biochemistry Test**	**Sample Number**	**Plate Culture**		**Biochemistry Test**
			**Plate 1**	**Plate 2**			**Plate 1**	**Plate 2**			**Plate 1**	**Plate 2**	
Water	*E. coli*	1	+	+	−	1	+	+	+	1	+	+	+
		2	+	+	+	2	+	+	+	2	+	+	−
		3	+	+	+	3	+	+	−	3	+	+	−
	*V. cholerae*	1	+	+	−	1	+	+	−	1	+	+	−
		2	−	−	NT	2	+	+	−	2	+	+	−
		3	+	−	+	3	+	+	−	3	+	+	−

Note: + means positive. − means negative. NT means note tested.

**Table 3:** Status of bacterial contamination in fish blood samples before and after the challenge.

**Before the Challenge**													
**Sample**	**Bacteria Strain**	**T1 (Control)**				**T2 (Challenged with *E. coli)***				**T3 (Challenged with *V. cholerae*)**			
		**Tank**	**Plate Culture**		**Biochemistry Test**	**Sample Number**	**Plate Culture**		**Biochemistry Test**	**Sample Number**	**Plate Culture**		**Biochemistry Test**
			**Plate 1**	**Plate 2**			**Plate 1**	**Plate 2**			**Plate 1**	**Plate 2**	
Blood	*E. coli*	1	−	−	NT	1	−	−	NT	1	−	−	NT
		2	−	−	NT	2	−	−	NT	2	−	−	NT
		3	−	−	NT	3	−	−	NT	3	−	−	NT
	*V. cholerae*	1	−	−	NT	1	−	−	NT	1	−	−	NT
		2	−	−	NT	2	−	−	NT	2	−	−	NT
		3	−	−	NT	3	−	−	NT	3	−	−	NT
**After the Challenge**													
**Sample**	**Bacteria Strain**	**T1 (Control)**				**T2 (Challenged with *E. coli*)**				**T3 (Challenged with *V. cholerae*)**			
		**Tank**	**Plate Culture**		**Biochemistry Test**	**Sample Number**	**Plate Culture**		**Biochemistry Test**	**Sample Number**	**Plate culture**		**Biochemistry Test**
			**Plate 1**	**Plate 2**			**Plate 1**	**Plate 2**			**Plate 1**	**Plate 2**	
Blood	*E. coli*	1	−	−	NT	1	−	−	NT	1	−	−	NT
		2	−	−	NT	2	−	−	NT	2	−	−	NT
		3	−	−	NT	3	−	−	NT	3	+	+	NT
	*V* *. cholerae*	1	−	−	NT	1	−	−	NT	1	+	+	−
		2	−	−	NT	2	−	−	NT	2	+	+	−
		3	−	−	NT	3	−	−	NT	3	−	−	NT

Note: + means positive. − means negative. NT means note tested.

**Table 4:** Status of bacterial contamination in fish feces samples before and after the challenge.

**Before the Challenge**													
**Sample**	**Bacteria Strain**	**T1 (Control)**				**T2 (Challenged with *E. coli*)**				**T3 (Challenged with *V. cholerae*)**			
		**Tank**	**Plate Culture**		**Biochemistry Test**	**Sample Number**	**Plate Culture**		**Biochemistry Test**	**Sample Number**	**Plate Culture**		**Biochemistry Test**
			**Plate 1**	**Plate 2**			**Plate 1**	**Plate 2**			**Plate 1**	**Plate 2**	
Feces	*E. coli*	1	+	+	+	1	−	−	NT	1	+	+	+
		2	+	−	−	2	+	+	+	2	+	+	−
		3	−	+	−	3	+	+	−	3	−	−	NT
	*V. cholerae*	1	+	−	−	1	−	−	NT	1	−	−	NT
		2	−	−	NT	2	+	+	−	2	−	−	NT
		3	+	+	+	3	−	−	NT	3	+	+	−
**After the Challenge**													
**Sample**	**Bacteria Strain**	**T1 (Control)**				**T2 (Challenged with *E. coli*)**				**T3 (Challenged with *V. cholerae*)**			
		**Tank**	**Plate Culture**		**Biochemistry Test**	**Sample Number**	**Plate Culture**		**Biochemistry Test**	**Sample Number**	**Plate Culture**		**Biochemistry Test**
			**Plate 1**	**Plate 2**			**Plate 1**	**Plate 2**			**Plate 1**	**Plate 2**	
Feces	*E. coli*	1	+	+	+	1	+	+	+	1	+	+	+
		2	+	+	+	2	+	+	−	2	+	+	+
		3	+	+	−	3	+	+	−	3	+	+	−
	*V. cholerae*	1	+	+	−	1	−	−	NT	1	+	+	−
		2	−	−	NT	2	+	+	−	2	+	+	−
		3	+	+	+	3	−	−	NT	3	+	+	−

Note: + means positive. − means negative. NT means note tested.

**Table 5:** Status of bacterial contamination in lettuce leaf samples before and after the challenge.

**Before the Challenge**													
**Sample**	**Bacteria Strain**	**T1 (Control)**				**T2 (Challenged with *E. coli*)**				**T3 (Challenged with *V. cholerae*)**			
		**Tank**	**Plate Culture**		**Biochemistry Test**	**Sample Number**	**Plate Culture**		**Biochemistry Test**	**Sample Number**	**Plate Culture**		**Biochemistry Test**
			**Plate 1**	**Plate 2**			**Plate 1**	**Plate 2**			**Plate 1**	**Plate 2**	
Leaves	*E. coli*	1	+	+	−	1	−	−	NT	1	+	+	−
		2	−	−	NT	2	+	−	−	2	−	−	NT
		3	−	−	NT	3	−	−	NT	3	−	−	NT
	*V. cholerae*	1	−	−	NT	1	−	−	NT	1	−	−	NT
		2	−	−	NT	2	+	+	−	2	−	−	NT
		3	+	+	+	3	+	+	−	3	+	−	−
**After the Challenge**													
**Sample**	**Bacteria Strain**	**T1 (Control)**				**T2 (Challenged with *E. coli*)**				**T3 (Challenged with *V. cholerae*)**			
		**Tank**	**Plate Culture**		**Biochemistry Test**	**Sample Number**	**Plate Culture**		**Biochemistry Test**	**Sample Number**	**Plate Culture**		**Biochemistry Test**
			**Plate 1**	**Plate 2**			**Plate 1**	**Plate 2**			**Plate 1**	**Plate 2**	
Leaves	*E. coli*	1	+	+	−	1	+	+	−	1	+	+	−
		2	+	−	−	2	+	+	+	2	−	−	NT
		3	−	−	NT	3	+	+	+	3	−	−	NT
	*V.* *cholerae*	1	+	+	−	1	−	−	NT	1	+	+	−
		2	−	−	NT	2	+	+	−	2	+	+	−
		3	+	−	+	3	+	+	−	3	+	+	+

Note: + means positive. − means negative. NT means note tested.

**Table 6:** Species-specific identification by molecular methods.

Sample	Treatment	Tank	Agar Type	Bacterium Species
Before the challenge				
Feces	T1	1	EMB	*C. freundii*
Feces	T1	3	TCBS	*Aeromonas* sp.
Feces	T2	2	EMB	*C. freundii*
Feces	T3	1	EMB	*C. freundii*
Lettuce leaves	T1	3	TCBS	*Aeromonas caviae*
After the challenge				
Water	T1	2, 3	EMB	*C. freundii*
Water	T1	3	TCBS	*Aeromonas* sp.
Water	T2	1, 2	EMB	*C. freundii*
Water	T3	1	EMB	*C. freundii*
Feces	T1	1, 2	EMB	Unidentified
Feces	T1	3	TCBS	*Aeromonas* sp.
Feces	T2	1	EMB	*Enterobacter hormaechei*
Feces	T3	1, 2	EMB	*C. freundii*
Feces	T3	1	TCBS	*C. freundii*
Lettuce leaves	T1	3	TCBS	*Aeromonas caviae*
Lettuce leaves	T2	2, 3	EMB	*C. freundii*
Lettuce leaves	T3	3	TCBS	*Aeromonas veronni*

## Data Availability

Data is contained within the article or Supplementary Materials.

## Supplementary Materials

The following supporting information can be downloaded at: https://www.mdpi.com/article/10.3390/biology15070559/s1, File S1. Contingency tables (Table S1. Contingency table used for Fisher’s exact test showing the presence or absence of *Escherichia coli* in feces collected before Nile tilapia were challenged with *E. coli* in the aquaponic system. The table summarizes the number of samples testing positive (present) or negative (absent) across the three treatments. Fisher’s exact test indicated no significant difference among treatments (*p* = 1.0000). Table S2. Contingency table used for Fisher’s exact test showing the presence or absence of *Escherichia coli* in lettuce leaves collected before Nile tilapia were challenged with *E. coli* in the aquaponic system. The table summarizes the number of samples testing positive (present) or negative (absent) across the three treatments. Fisher’s exact test indicated no significant difference among treatments (*p* = 1.0000). Table S3. Contingency table used for Fisher’s exact test showing the presence or absence of *Vibrio cholerae* in water collected before Nile tilapia were challenged with *V. cholerae* in the aquaponic system. The table summarizes the number of samples testing positive (present) or negative (absent) across the three treatments. Fisher’s exact test indicated no significant difference among treatments (*p* = 0.6786). Table S4. Contingency table used for Fisher’s exact test showing the presence or absence of *Vibrio cholerae* in feces collected before Nile tilapia were challenged with *V. cholerae* in the aquaponic system. The table summarizes the number of samples testing positive (present) or negative (absent) across the three treatments. Fisher’s exact test indicated no significant difference among treatments (*p* = 1.0000). Table S5. Contingency table used for Fisher’s exact test showing the presence or absence of *Vibrio cholerae* in lettuce leaves collected before Nile tilapia were challenged with *V. cholerae* in the aquaponic system. The table summarizes the number of samples testing positive (present) or negative (absent) across the three treatments. Fisher’s exact test indicated no significant difference among treatments (*p* = 1.0000). Table S6. Contingency table used for Fisher’s exact test showing the presence or absence of *Escherichia coli* in lettuce leaves collected after Nile tilapia were challenged with *E. coli* in the aquaponic system. The table summarizes the number of samples testing positive (present) or negative (absent) across the three treatments. Fisher’s exact test indicated no significant difference among treatments (*p* = 0.6786). Table S7. Contingency table used for Fisher’s exact test showing the presence or absence of *Vibrio cholerae* in water collected after Nile tilapia were challenged with *V. cholerae* in the aquaponic system. The table summarizes the number of samples testing positive (present) or negative (absent) across the three treatments. Fisher’s exact test indicated no significant difference among treatments (*p* = 1.0000). Table S8. Contingency table used for Fisher’s exact test showing the presence or absence of *Vibrio cholerae* in feces collected after Nile tilapia were challenged with *V. cholerae* in the aquaponic system. The table summarizes the number of samples testing positive (present) or negative (absent) across the three treatments. Fisher’s exact test indicated no significant difference among treatments (*p* = 0.6786). Table S9. Contingency table used for Fisher’s exact test showing the presence or absence of *Vibrio cholerae* in lettuce leaves collected after Nile tilapia were challenged with *V. cholerae* in the aquaponic system. The table summarizes the number of samples testing positive (present) or negative (absent) across the three treatments. Fisher’s exact test indicated no significant difference among treatments (*p* = 1.0000)). Figure S1: Results of microbiological analysis of samples from disinfection processes. (a) EMB for *E. coli*. (b) TCBS for *V. cholerae*. (c) TSA—non-selective agar. Figure S2: Presumptive colonies results. (a) EMB for *E. coli* and (b) TCBS for *V. cholerae.* Figure S3: Results of biochemistry tests. (a) negative for *E. coli* in TSI agar, (b) positive for *E. coli* in TSI, and (c) positive and negative for *V. cholerae* in oxidase test. File S2: Laboratory Certificate for sequencing analysis results. File S3: Ethical approval Certificate.
