# Supplementary material for: Detection of Bacterial Internalization in Lettuce (Lactuca sativa) Leaves Grown in Aquaponic Systems with Nile Tilapia (Oreochromis niloticus) Under Microbial Challenge
Source: Biology (Basel). 2026 Mar 31;15(7):559. doi: 10.3390/biology15070559 (PMC13072089; doi:10.3390/biology15070559)
Supplement: Supplementary file 1 [file biology-15-00559-s001.zip › File S1 Contingency tables.pdf]

Escherichia coli **before challenge: Feces**

Table S1. Contingency table used for Fisher's exact test showing the presence or absence of *Escherichia coli* in feces collected before Nile tilapia were challenged with *E. coli* in the aquaponic system. The table summarizes the number of samples testing positive (present) or negative (absent) across the three treatments. Fisher's exact test indicated no significant difference among treatments ($p = 1.0000$).

**Treatment**	**Result**	
	**Absent**	**Present**
Treatment 1	0	3
Treatment 2	1	2
Treatment 3	1	2
p-value	1.0000	

Escherichia coli **before challenge: Lettuce leaves**

Table S2. Contingency table used for Fisher's exact test showing the presence or absence of *Escherichia coli* in lettuce leaves collected before Nile tilapia were challenged with *E. coli* in the aquaponic system. The table summarizes the number of samples testing positive (present) or negative (absent) across the three treatments. Fisher's exact test indicated no significant difference among treatments ($p = 1.0000$).

**Treatment**	**Result**	
	**Absent**	**Present**
Treatment 1	2	1
Treatment 2	2	1
Treatment 3	2	1
p-value	1.0000	

Vibrio cholerae **before challenge: water**

Table S3. Contingency table used for Fisher's exact test showing the presence or absence of *Vibrio cholerae* in water collected before Nile tilapia were challenged with *V. cholerae* in the aquaponic system. The table summarizes the number of samples testing positive (present) or negative (absent) across the three treatments. Fisher's exact test indicated no significant difference among treatments ($p = 0.6786$).

**Treatment**	**Result**	
	**Absent**	**Present**
Treatment 1	2	1
Treatment 2	1	2
Treatment 3	0	3
p-value	0.6786	

Vibrio cholerae **before challenge: feces**

Table S4. Contingency table used for Fisher's exact test showing the presence or absence of *Vibrio cholerae* in feces collected before Nile tilapia were challenged with *V. cholerae* in the aquaponic system. The table

summarizes the number of samples testing positive (present) or negative (absent) across the three treatments. Fisher's exact test indicated no significant difference among treatments (p = 1.0000).

**Treatment**	**Result**	
	**Absent**	**Present**
Treatment 1	1	2
Treatment 2	2	1
Treatment 3	2	1
p-value	1.0000	

***Vibrio cholerae* before challenge: Lettuce leaves**

Table S5. Contingency table used for Fisher's exact test showing the presence or absence of *Vibrio cholerae* in lettuce leaves collected before Nile tilapia were challenged with *V. cholerae* in the aquaponic system. The table summarizes the number of samples testing positive (present) or negative (absent) across the three treatments. Fisher's exact test indicated no significant difference among treatments (p = 1.0000).

**Treatment**	**Result**	
	**Absent**	**Present**
Treatment 1	2	1
Treatment 2	1	2
Treatment 3	2	1
p-value	1.0000	

***Escherichia coli* after challenge: Lettuce leaves**

Table S6. Contingency table used for Fisher's exact test showing the presence or absence of *Escherichia coli* in lettuce leaves collected after Nile tilapia were challenged with *E. coli* in the aquaponic system. The table summarizes the number of samples testing positive (present) or negative (absent) across the three treatments. Fisher's exact test indicated no significant difference among treatments (p = 0.6786).

**Treatment**	**Result**	
	**Absent**	**Present**
Treatment 1	1	2
Treatment 2	0	3
Treatment 3	2	1
p-value	0.6786	

***Vibrio cholerae* after challenge: water**

Table S7. Contingency table used for Fisher's exact test showing the presence or absence of *Vibrio cholerae* in water collected after Nile tilapia were challenged with *V. cholerae* in the aquaponic system. The table summarizes the number of samples testing positive (present) or negative (absent) across the three treatments. Fisher's exact test indicated no significant difference among treatments (p = 1.0000).

Treatment	Result	
	**Absent**	**Present**
Treatment 1	2	1
Treatment 2	3	0
Treatment 3	3	0
p-Value	1.0000	

***Vibrio cholerae* after challenge: feces**

Table S8. Contingency table used for Fisher's exact test showing the presence or absence of *Vibrio cholerae* in feces collected after Nile tilapia were challenged with *V. cholerae* in the aquaponic system. The table summarizes the number of samples testing positive (present) or negative (absent) across the three treatments. Fisher's exact test indicated no significant difference among treatments ($p = 0.6786$).

Treatment	Result	
	**Absent**	**Present**
Treatment 1	1	2
Treatment 2	2	1
Treatment 3	0	3
p-Value	0.6786	

***Vibrio cholerae* after challenge: Lettuce leaves**

Table S9. Contingency table used for Fisher's exact test showing the presence or absence of *Vibrio cholerae* in lettuce leaves collected after Nile tilapia were challenged with *V. cholerae* in the aquaponic system. The table summarizes the number of samples testing positive (present) or negative (absent) across the three treatments. Fisher's exact test indicated no significant difference among treatments ($p = 1.0000$).

Treatment	Result	
	**Absent**	**Present**
Treatment 1	1	2
Treatment 2	1	2
Treatment 3	0	3
p-Value	1.0000	
