# Supplementary material for: Detection of Bacterial Internalization in Lettuce (Lactuca sativa) Leaves Grown in Aquaponic Systems with Nile Tilapia (Oreochromis niloticus) Under Microbial Challenge
Source: Biology (Basel). 2026 Mar 31;15(7):559. doi: 10.3390/biology15070559 (PMC13072089; doi:10.3390/biology15070559)
Supplement: Supplementary file 1 [file biology-15-00559-s001.zip › File S2 File Laboratory Certificate for sequencing analysis results.pdf]

ESALQ USP

LABMIC Biomol e Micotoxinas

DEPARTMENT OF

FOOD SCIENCE AND TECHNOLOGY

Av. Pádua Dias, 11 ◆ P.O. Box 9 ◆ CEP13418-900 ◆ Piracicaba, SP ◆ Brazil

Telephone: (19) 3447-6007 or 3447-6009 – email: labmic@usp.br

CERTIFICATE OF ANALYSIS

(Translated from the original in Portuguese (attached) into English)

**Customer:**	**Angélica Adiação Jossefa**
**Analysis Performed:**	**Bacterial species identification**
**Sample description:**	**Unspecified bacterial culture**
**Quantity supplied:**	**not applicable**
**Date of receipt:**	**30/ 07/ 2025**
**Certificate number:**	**2025/11-03**

RESULTS:

The species were identified according to the methodology of Crossley, B.M. *et al.*, 2020.

Two sequencing operations were performed for sample T3LE1, which confirmed the same results, that is, they belong to different species. Sequence F showed 81.35% identity with the species *Enterobacter mori*, while sequence R showed 96.11% identity with the species *Citrobacter freundii*.

The results for each sample are shown below.

Translator's Certification:

"I, Clésio José Machava, the undersigned sworn translator, hereby certify that this translation is a true and accurate rendering of the original document in Portuguese attached to this certificate."

Registration No.: **P43 of 19/06/2009;** Date of Translation: **20/01/2026**

Signature and stamp: Clésio Machava

CLÉSIO J. MACHAVA
Sworn Translator
PORTUGUESE / ENGLISH
NUIT: 101092038
E-mail: clesio.machava@gmail.com

DEPARTAMENTO DE
CIÊNCIA E TECNOLOGIA DE ALIMENTOS
Av. Pádua Dias, 11 • Caixa Postal 9 • CEP 13418-900 • Piracicaba, SP • Brasil
Telefone: (19) 3447-6007 ou 3447-6009 – e-mail: labmic@usp.br

CERTIFICADO DE ANÁLISE

Cliente: **Angélica Adiação Jossefa**

Análise realizada: **Identificação de espécie de bactéria**

Descrição da amostra: **Cultura bacteriana não especificada**

Quantidade fornecida: **Não aplicável**

Data de recebimento: **30/07/2025**

Número do certificado: **2025/11-03**

RESULTADOS:

As espécies foram identificadas segundo a metodologia de Crossley, B.M. et al., 2020.

Foram realizados dois sequenciamentos para a amostra T3LE1, os quais confirmaram os mesmos resultados, ou seja, pertencem a espécies distintas. A sequência F apresentou 81,35% de identidade com a espécie *Enterobacter mori*, já a sequência R apresentou 96,11% de identidade com a espécie *Citrobacter freundii*.

Segue abaixo os resultados para cada amostra

ESALQ USP

DEPARTAMENTO DE
CIÊNCIA E TECNOLOGIA DE ALIMENTOS

LABMIC Biomol e Micotoxinas

Av. Pádua Dias, 11 • Caixa Postal 9 • CEP 13418-900 • Piracicaba, SP • Brasil
Telefone: (19) 3447-6007 ou 3447-6009 – e-mail: labmic@usp.br

Amostra	Sequência F	Sequência R	Nome Científico
T1HE1	GCCTACACATGCAGTCGA<br>ACGGTAGCACAGAGGAGC<br>TTGCTCCTTGGGTGACGA<br>GTGGCGGACSGGTGAGTA<br>ATGTCTGGGAAACTGCCC<br>GATGGAGGGGGATAACTA<br>CTGGAAACGGTAGCTAATA<br>CCGCATAACGTCGCAAGA<br>CCAAAGAGGGGGACCTTC<br>GGGCCTCTTGCCATCGGA<br>TGTGCCCAGATGGGATTA<br>GCTAGTAGGTGGGGTAAC<br>GGCTCACCTAGGCGACGA<br>TCCCTAGCTGGTCTGAGA<br>GGATGACCAGCCACACTG<br>GAACTGAGACACGGTCCA<br>GACTCCTACGGGAGGCAG<br>CAGTGGGGAATATTGCAC<br>AATGGGCGCAAGCCTGAT<br>GCAGCCATGCCGCGTGTA<br>TGAAGAAGGCCTTCGGGT<br>TGTAAAGTACTTTCAGCGA<br>GGAGGAAGGCGTTGTGGT<br>TAATAACCGCAGCGATTGA<br>CGTTACTCGCAGAAGAAG<br>CACCGGCTAACTCCGTGC<br>CAGCAGCCGCGGTAAA	TGCGAGTACGTCAATCGCTG<br>CGGTTATTAACCTCAACGCC<br>TTCCTCCTCGCTGAAAGTAC<br>TTTACAACCCGAAGGCCTTC<br>TTCATACACGCGGCATGGCT<br>GCATCAGGCTTGCTCCCATT<br>GTGCAATATTCCCCACTGCT<br>GCCTCCCGTAGGAGTCTGG<br>ACCGTGTCTCAGTTCCAGTG<br>TGGCTGGTCATCCTCTCAGA<br>CCAGCTAGGGATCGTCGCCT<br>AGGTGAGCCGTTACCCCACC<br>TACTAGCTAATCCCATCTGGG<br>CACATCCGATGGCAAGAGGC<br>CCGAAGGTCCCCCTCTTTGG<br>TCTTGCGACGTTATGCGGTA<br>TTAGCTACCGTTTCCAGTAGT<br>TATCCCCCTCCATCGGGCAG<br>TTTCCCAGACATTACTCACC<br>CGTCCGCCACTCGTCACCC<br>AAGGAGCAAGCTCCTCTGTG<br>CTACCGTTCGACTTGCATGT<br>GTTAGGCCTGCCGCCAGCG<br>TTCAATCTGAGCCATGATCAA<br>ACTCA	Citrobacter freundii

ESALQ USP

DEPARTAMENTO DE
CIÊNCIA E TECNOLOGIA DE ALIMENTOS

LABMIC Biomol e Micotoxinas

Av. Pádua Dias, 11 • Caixa Postal 9 • CEP 13418-900 • Piracicaba, SP • Brasil
Telefone: (19) 3447-6007 ou 3447-6009 – e-mail: labmic@usp.br

Amostra	Sequência F	Sequência R	Nome Científico
T1HE2	GCAAGTCGAACGGTAGCA<br>CAGAGGAGCTTGCTCCTT<br>GGGTGACGAGTGGCGGA<br>CGGGTGAGTAATGTCTGG<br>GAAACTGCCCGATGGAGG<br>GGGATAACTACTGGAAAC<br>GGTAGCTAATACCGCATAA<br>CGTCGCAAGACCAAAGAG<br>GGGGACCTTCGGGCCTCT<br>TGCCATCGGATGTGCCCA<br>GATGGGATTAGCTAGTAGG<br>TGGGGTAACGGCTCACCT<br>AGGCGACGATCCCTAGCT<br>GGTCTGAGAGGATGACCA<br>GCCACACTGGAACTGAGA<br>CACGGTCCAGACTCCTAC<br>GGGAGGCAGCAGTGGGG<br>AATATTGCACAATGGGCGC<br>AAGCCTGATGCAGCCATG<br>CCGCGTGGATGAAGAAGG<br>CCTTCGGGTTGTAAAGTAC<br>TTTCAGCGAGGAGGAAGG<br>TGTTGTGGTTAATAACCGC<br>AGCGATTGACGTTACTCG<br>CAGAAGAAGCACCGGCTA<br>ACTCCGTGCCAGCAGCCG<br>CGGTAAA	AGTACGTCATTGCTGCG<br>GTTATTAACCACAACGC<br>CTTCCTCCTCGCTGAAA<br>GTACTTTACAACCCGAA<br>GGCCTTCTTCATACACG<br>CGGCATGGCTGCATCA<br>GGCTTGCGCCCATTGT<br>GCAATATTCCCCACTGC<br>TGCCTCCCGTAGGAGT<br>CTGGACCGTGTCTCAG<br>TTCCAGTGTGGCTGGT<br>CATCCTCTCAGACCAGC<br>TAGGGATCGTCGCCTAG<br>GTGAGCCGTTACCCCA<br>CCTACTAGCTAATCCCA<br>TCTGGGCACATCCGATG<br>GCAAGAGGCCCGAAGG<br>TCCCCCTCTTTGGTCTT<br>GCGACGTTATGCGGTAT<br>TAGCTACCGTTTCCAGT<br>AGTTATCCCCCTCCATC<br>GGGCAGTTTCCCAGAC<br>ATTACTCACCCGTCCGC<br>CACTCGTCACCCAAGG<br>AGCAAGCTCCTCTGTG<br>CTACCGTTCGACTTGCA<br>TGTGTTAGGCCTGCCG<br>CCAGCGTTCAATCTGAG<br>CAAGGATCAAACTCA	Citrobacter freundii

DEPARTAMENTO DE
CIÊNCIA E TECNOLOGIA DE ALIMENTOS
LABMIC Biomol e Micotoxinas
Av. Pádua Dias, 11 • Caixa Postal 9 • CEP 13418-900 • Piracicaba, SP • Brasil
Telefone: (19) 3447-6007 ou 3447-6009 – e-mail: labmic@usp.br

Amostra	Sequência F	Sequência R	Nome Científico
T1HE3	ATGCAAGTCGAACGGTA<br>GCACAGAGGAGCTTGCT<br>CCTTGGGTGACGAGTGG<br>CGGACGGGTGAGTAATG<br>TCTGGGAAACTGCCCGA<br>TGGAGGGGGATAACTAC<br>TGGAAACGGTAGCTAATA<br>CCGCATAACGTCGCAAG<br>ACCAAAGAGGGGGACCT<br>TCGGGCCTCTTGCCATC<br>GGATGTGCCCAGATGGG<br>ATTAGCTAGTAGGTGGG<br>GTAACGGCTCACCTAGG<br>CGACGATCCCTAGCTGG<br>TCTGAGAGGATGACCAG<br>CCACACTGGAACTGAGA<br>CACGGTCCAGACTCCTA<br>CGGGAGGCAGCAGTGG<br>GGAATATTGCACAATGG<br>GCGCAAGCCTGATGCAG<br>CCATGCCGCGTGTATGA<br>AGAAGGCCTTCGGGTTG<br>TAAAGTACTTTCAGCGAG<br>GAGGAAGGTGTTGTGGT<br>TAATAACCGCAGCGATTG<br>ACGTTACTCGCAGAAGA<br>AGCACCGGCTAACTCCG<br>TGCCAGCAGCCGCGGTA<br>AA	TGCGAGTACGTCATTGCT<br>GCGGTTATTAACCACAACG<br>CCTTCCTCCTCGCTGAAA<br>GTACTTTACAACCCGAAG<br>GCCTTCTTCATACACGCG<br>GCATGGCTGCATCAGGCT<br>TGCGCCCATTGTGCAATAT<br>TCCCCACTGCTGCCTCCC<br>GTAGGAGTCTGGACCGTG<br>TCTCAGTTCCAGTGTGGC<br>TGGTCATCCTCTCAGACC<br>AGCTAGGGATCGTCGCCT<br>AGGTGAGCCGTTACCCCA<br>CCTACTAGCTAATCCCATC<br>TGGGCACATCCGATGGCA<br>AGAGGCCCGAAGGTCCC<br>CCTCTTTGGTCTTGCGAC<br>GTTATGCGGTATTAGCTAC<br>CGTTTCCAGTAGTTATCCC<br>CCTCCATCGGGCAGTTTC<br>CCAGACATTACTCACCCGT<br>CCGCCACTCGTCACCCAA<br>GGAGCAAGCTCCTCTGTG<br>CTACCGTTCGACTTGCATG<br>TGTTAGGCCTGCCGCCAG<br>CGTTCAATCTGAGC	Citrobacter freundii

DEPARTAMENTO DE
CIÊNCIA E TECNOLOGIA DE ALIMENTOS
Av. Pádua Dias, 11 • Caixa Postal 9 • CEP 13418-900 • Piracicaba, SP • Brasil
Telefone: (19) 3447-6007 ou 3447-6009 – e-mail: labmic@usp.br

Amostra	Sequência F	Sequência R	Nome Científico
T2HE1	CAGTCGAACGGTAGCAC AGAGGAGCTTGSTCCTT GGGTGACGAGTGGCGG ACGGGTGAGTAATGTCT GGGAAACTGCCCGATGG AGGGGGATAACTACTGG AAACGGTAGCTAATACCG CATAACGTCGCAAGACC AAAGAGGGGGACCTTCG GGCCTCTTGCCATCGGA TGTGCCCAGATGGGATT AGCTAGTAGGTGGGGTA ACGGCTCACCTAGGCGA CGATCCCTAGCTGGGCT GAGAGGATGACCAGCCA CACTGGAACTGAGACAC GGTCCAGACTCCTACGG GAGGCAGCAGTGGGGA ATATTGCACAATGGGCGC AAGCCTGATGCAGCCAT GCCGCGTGTATGAAGAA GGCCTTCGGGTTGTAAA GTACTTTCAGCGAGGAG GAAGGCGTTGGGGTTAA TAACCGCAGCGATTGAC GTTACTCGCAGAAGAAG CACCGGCTAACTCCGTG CCAGCAGCCGCGGTAAA	CGTCAATCGCTGCGGTTAT TAACCACAACGCCTTCCTC CGCGCTGAAAGTACTTTAC AACCCGAAGGCCTTCTTCA TACACGCGGCATGGCTGCA TCAGGCTTGCGCCCATTGT GCAATATTCCCCACTGCTG CCTCCCGTAGGAGTCTGGA CCGTGTCTCAGTTCCAGTG TGGCTGGTCATCCTCTCAG ACCAGCTAGGGATCGTCGC CTAGGTGAGCCGTTACCCC ACCTACTAGCTAATCCCATC TGGGCACATCCGATGGCAA GAGGCCCGAAGGTCCCCC TCTTTGGTCTTGCGACGTT ATGCGGTATTAGCTACCGTT TCCAGTAGTTATCCCCCTC CATCGGGCAGTTTCCCAGA CATTACTCACCCGTCCGCC GCTCGTCACCCAAGGAGC AAGCTCCTCTGTGCTACCG TTCGACTTGCATGTGTTAG GCCTGCCGCCAGCGTTCA ATCTGA	**Citrobacter freundii**

ESALQ USP

DEPARTAMENTO DE
CIÊNCIA E TECNOLOGIA DE ALIMENTOS

LABMIC Biomol e Micotoxinas

Av. Pádua Dias, 11 • Caixa Postal 9 • CEP 13418-900 • Piracicaba, SP • Brasil
Telefone: (19) 3447-6007 ou 3447-6009 – e-mail: labmic@usp.br

Amostra	Sequência F	Sequência R	Nome Científico
T2HE2	ACATGCAAGTCGACGGT<br>AGCACAGAGGAGCTGG<br>CTCCTTGGGTGACGAG<br>TGGCGGACGGGTGAGT<br>AGTGTCTGGGAAACTG<br>CCCGATGGAGGGGGAT<br>AACTACTGGAAACGGTA<br>GCTAATACCGCATAACG<br>TCGCAAGACCAAAGAG<br>GGGGACCTTCGGGCCT<br>CTTGCCATCGGATGTGC<br>CCAGATGGGATTAGCTA<br>GTAGGTGGGGTAACGG<br>CTCACCTAGGCGACGAT<br>CCCTAGCTGGTCTGAG<br>AGGATGACCASCCACA<br>CTGGAACTGAGACACG<br>GTCCAGACTCCTACGG<br>GAGGCAGCAGTGGGGA<br>ATAT TGCACAGTGGGCG<br>CAAGCCTGATGCASCC<br>GTGCCGCGTGTATGAA<br>GAAGGCCTTCGGGTTG<br>TAAAGTACTTTCAGCGA<br>GGAGGAAGGCGTTGTG<br>GTTAATAACCGCAGCGA<br>T TGACGTTACTCGCAGA<br>AGAAGCACCGGCTAAC<br>TCCGTGCCAG	TCTGCGAGTACGTCATCG<br>CTGCGGTTATTAACCACAA<br>CGCCTTCCTCCCCGCTGA<br>AAGTACTTTACAACCCGAA<br>GGCCTTCTTCATACACGC<br>GGCATGGCTGCATCAGGC<br>TTGCGCCCATTGTGCAATA<br>TTCCCCACTGCTGCCTCC<br>CGTAGGAGTCTGGACCGT<br>GTCTCAGTTCCAGTGTGG<br>CTGGTCATCCTCTCAGAC<br>CAGCTAGGGATCGTCGCC<br>TAGGTGAGCCGTTACCCC<br>ACCTACTAGCTAATCCCAT<br>CTGGGCACATCCGATGGC<br>AAGAGGCCCGAAGGTCCC<br>CCTCTTTGGTCTTGCGAC<br>GTTATGCGGTATTAGCTAC<br>CGTTTCCAGTAGTTATCCC<br>CCTCCATCRGGCAGTTTC<br>CCAGACATTACTCACCCGT<br>CCGCCACTCGTCACCCAA<br>GGAGAAAGCTCCTCTGTG<br>CTACCGTTCGACTTGCGT<br>GTGTTAGGCCTGCCSCCA<br>GCGTTCAATCTGAGCCAG<br>GATCAAACTCTAAA	**Citrobacter freundii**

DEPARTAMENTO DE
CIÊNCIA E TECNOLOGIA DE ALIMENTOS

Av. Pádua Dias, 11 • Caixa Postal 9 • CEP 13418-900 • Piracicaba, SP • Brasil
Telefone: (19) 3447-6007 ou 3447-6009 – e-mail: labmic@usp.br

Amostra	Sequência F	Sequência R	Nome Científico
T3FV2	CACATGCAAGTCGAGC<br>GGCAGCGGGAAAGTAG<br>CTTGCTACTTTTGCCGG<br>CGAGCGGCGGACGGGT<br>GAGTAATGCCTGGGAAA<br>TTGCCCAGTCGAGGGG<br>GATAACAGTTGGAAACG<br>ACTGCTAATACCGCATA<br>CGCCCTACGGGGGAAA<br>GCAGGGGACCTTCGGG<br>CCTTGCGCGATTGGATA<br>TGCCCAGGTGGGATTA<br>GCTAGTTGGTGAGGTAA<br>TGGCTCACCAAGGCGA<br>CGATCCCTAGCTGGTCT<br>GAGAGGATGATCAGCC<br>ACACTGGAACTGAGAC<br>ACGGTCCAGACTCCTAC<br>GGGAGGCAGCAGTGGG<br>GAATATTGCACAATGGG<br>GGAAACCCTGATGCAG<br>CCATGCCGCGTGTGTG<br>AAGAAGGCCTTCGGGT<br>TGTAAAGCACTTTCAGC<br>GAGGAGGAAAGGTCAG<br>TAGCTAATATCTGCTGG<br>CTGTGACGTTACTCGCA<br>RAAGAAGCACCGGCTA<br>ACTCCGTGCCA	AGTACGTCACAGCCASCA<br>GATATTAGCTACTGACCTT<br>TCCTCCTCGCTGAAAGTG<br>CTTTACAACCCGAAGGCC<br>TTCTTCACACACGCGGCA<br>TGGCTGCATCAGGGTTTC<br>CCCCATTGTGCAATATTCC<br>CCACTGCTGCCTCCCGTA<br>GGAGTCTGGACCGTGTCT<br>CAGTTCCAGTGTGGCTGA<br>TCATCCTCTCARACCAGCT<br>AGGGATCGTCGCCTTGGT<br>GAGCCATTACCTCACCAA<br>CTAGCTAATCCCACCTGG<br>GCATATCCAATCGCGCAA<br>GGCCCGAAGGTCCCCTG<br>CTTTCCCCCGTAGGGCGT<br>ATGCGGTATTAGCAGTCGT<br>TTCCAACTGTTATCCCCCT<br>CGACTGGGCAATTTCCCA<br>GGCATTACTCACCCGTCC<br>GCCGCTCGCCGGCAAAA<br>GTAGCAAGCTACTTTCCC<br>GCTGCCGCTCGACTTGCA<br>TGTGTTAGGCCTGCCGCC<br>AGCGTTCAATCTGAG	**Aeromonas caviae**

DEPARTAMENTO DE
CIÊNCIA E TECNOLOGIA DE ALIMENTOS
Av. Pádua Dias, 11 • Caixa Postal 9 • CEP 13418-900 • Piracicaba, SP • Brasil
Telefone: (19) 3447-6007 ou 3447-6009 – e-mail: labmic@usp.br

Amostra	Sequência F	Sequência R	Nome Científico
T3HE1	CTACACATGCAAGTCGA<br>ACGGTAGCACAGAGGA<br>GCTTGCTCCTTGGGTG<br>ACGAGTGGCGGACGGG<br>TGAGTAATGTCTGGGAA<br>ACTGCCCGATGGAGGG<br>GGATAACTACTGGAAAC<br>GGTAGCTAATACCGCAT<br>AACGTCGCAAGACCAA<br>AGAGGGGGACCTTCGG<br>GCCTCTTGCCATCGGAT<br>GTGCCCAGATGGGATTA<br>GCTAGTAGGTGGGGTAA<br>CGGCTCACCTAGGCGA<br>CGATCCCTAGCTGGTCT<br>GAGAGGATGACCAGCC<br>ACACTGGAACTGAGAC<br>ACGGTCCAGACTCCTAC<br>GGGAGGCAGCAGTGGG<br>GAATATTGCACAATGGG<br>CGCAAGCCTGATGCAG<br>CCATGCCGCGTGTATGA<br>AGAAGGCCTTCGGGTT<br>GTAAAGTACTTTCAGCG<br>AGGAGGAAGGCGTTGT<br>GGTTAATAACCGCRGCG<br>ATTGACGTTACTCGCAG<br>AAGAAGCACCGGCTAA<br>CTCCGTGCCAGCAGCC<br>GCGGTAAA	CMGGCCTCTTCTKCGAGT<br>ACGTCATCGCTGCGGTTAT<br>TAACCACAACGCCTTCCT<br>CCTCGCTGAAAGTACTTTA<br>CAACCCGAAGGCCTTCTT<br>CATACACGCGGCATGGCT<br>GCATCAGGCTTGCGCCCA<br>TTGTGCAATATTCCCCACT<br>GCTGCCTCCCGTAGGAGT<br>CTGGACCGTGTCTCAGTT<br>CCAGTGTGGCTGGTCATC<br>CTCTCAGACCAGCTAGGG<br>ATCGTCGCCTAGGTGAGC<br>CGTTACCCCACCTACTAGC<br>TAATCCCATCTGGGCACAT<br>CCGATGGCAAGAGGCCC<br>GAAGGTCCCCCTCTTTGG<br>TCTTGCGACGTTATGCGGT<br>ATTAGCTACCGTTTCCAGT<br>AGTTATCCCCCTCCATCGG<br>GCAGTTTCCCAGACATTAC<br>TCACCCGTCCGCCACTCG<br>TCACCCAAGGAGCAAGCT<br>CCTCTGTGCTACCGTTCG<br>ACTTGCATGTGTTAGGCCT<br>GCCGCCAGCGTTCAATCT<br>GAGCAAGGATCAAACTCA	**Citrobacter freundii**

ESALQ
USP

DEPARTAMENTO DE
CIÊNCIA E TECNOLOGIA DE ALIMENTOS

Av. Pádua Dias, 11 • Caixa Postal 9 • CEP 13418-900 • Piracicaba, SP • Brasil
Telefone: (19) 3447-6007 ou 3447-6009 – e-mail: labmic@usp.br

LABMIC Biomol e Micotoxinas

Amostra	Sequência F	Sequência R	Nome Científico
T1FE1	TACACATGCAAGTCGAA<br>CGGTAGCACAGAGGAG<br>CTTGCTCCTTGGGTGAC<br>GAGTGGCGGACGGGTG<br>AGTAATGTCTGGGAAAC<br>TGCCCGATGGAGGGGG<br>ATAACTACTGGAAACGG<br>TAGCTAATACCGCATAAC<br>GTCGCAAGACCAAAGA<br>GGGGGACCTTCGGGCC<br>TCTTGCCATCGGATGTG<br>CCCAGATGGGATTAGCT<br>AGTAGGTGGGGTAACG<br>GCTCACCTAGGCGACG<br>ATCCCTAGCTGGTCTGA<br>GAGGATGACCAGCCAC<br>ACTGGAACTGAGACAC<br>GGTCCAGACTCCTACG<br>GGAGGCAGCAGTGGGG<br>AATATTGCACAATGGGC<br>GCAAGCCTGATGCAGC<br>CATGCCGCGTGTATGAA<br>RAAGGCCTTCGGGTTG<br>TAAAGTACTTTCAGCGA<br>GGAGGAAGGCGTTGTG<br>GTTAATAACCGCAGCGA<br>TTGACGTTACTCGCAGA<br>AGAAGCACCGGCTAAC<br>TCCGTGCC	GTACGTCATCGCTGCGGT<br>TATTAACCACAACGCCTTC<br>CTCCCCGCTGAAAGTACT<br>TTACAACCCGAAGGCCTT<br>CTTCATACACGCGGCATG<br>GCTGCATCAGGCTTGCGC<br>CCATTGTGCAATATTCCCC<br>ACTGCTGCCTCCCGTAGG<br>AGTCTGGACCGTGTCTCA<br>GTTCCAGTGTGGCTGGTC<br>ATCCTCTCAGACCAGCTA<br>GGGATCGTCGCCTAGGTG<br>AGCCGTTACCCCACCTACT<br>AGCTAATCCCATCTGGGC<br>ACATCCGATGGCAAGAGG<br>CCCGAAGGTCCCCCTCTT<br>TGGTCTTGCGACGTTATG<br>CGGTATTAGCTACCGTTTC<br>CAGTAGTTATCCCCCTCCA<br>TCGGGCAGTTTCCCAGAC<br>ATTACTCACCCGTCCGCC<br>ACTCGTCACCCAAGGAGC<br>AAGCTCCTCTGTGCTACC<br>GTTCGACTTGCATGTGTTA<br>GGCCTGCCGCCAGCGTTC<br>AATCTGAG	**Citrobacter freundii**

ESALQ
USP

DEPARTAMENTO DE
CIÊNCIA E TECNOLOGIA DE ALIMENTOS

LABMIC
Biomol e Micotoxinas

Av. Pádua Dias, 11 • Caixa Postal 9 • CEP 13418-900 • Piracicaba, SP • Brasil
Telefone: (19) 3447-6007 ou 3447-6009 – e-mail: labmic@usp.br

Amostra	Sequência F	Sequência R	Nome Científico
T1FE2	ATGCAAGTCGAACGGTA<br>GCACAGAGGAGCTTGC<br>TCCTTGGGTGACGAGT<br>GGCGGACGGGTGAGTT<br>ATGTCTGGGAAACTGCC<br>CGATGGAGGGGGATAA<br>CTACTGGAAACGGTAGC<br>TAATACCGCATAACGTC<br>GCAAGACCAAAGAGGG<br>GGACCTTCGGGCCTCT<br>TGCCATCGGATGTGCC<br>CAGATGGGATTAGCTAG<br>TAGGTGGGGTAACGGC<br>TCACCTAGGCGACGATC<br>CCTAGCTGGTCTGAGA<br>GGATGACCAGCCACAC<br>TGGAACTGAGACACGG<br>TCCAGACTCCTACGGG<br>AGGCAGCAGTGGGGAA<br>TATTGCACAATGGGCGC<br>AAGCCTGATGCAGCCAT<br>GCCGCGTGTATGAAGA<br>AGGCCTTCGGGTTGTAA<br>AGTACTTTCAGCGAGGA<br>GGAAGGTGTTGTGGTTA<br>ATAACCGCAGCAATTGA<br>CGTTACTCGCAGAAGAA<br>GCACCGGCTAACTCCG<br>TGC	ACGTCAATTGCTGCGGTTA<br>TTAACCACAACACCTTCCT<br>CCTCGCTGAAAGTACTTTA<br>CAACCCGAAGGCCTTCTT<br>CATACACGCGGCATGGCT<br>GCATCAGGCTTGCGCCCA<br>TTGTGCAATATTCCCCACT<br>GCTGCCTCCCGTAGGAGT<br>CTGGACCGTGTCTCAGTT<br>CCAGTGTGGCTGGTCATC<br>CTCTCAGACCAGCTAGGG<br>ATCGTCGCCTAGGTGAGC<br>CGTTACCCCACCTACTAGC<br>TAATCCCATCTGGGCACAT<br>CCGATGGCAAGAGGCCC<br>GAAGGTCCCCCTCTTTGG<br>TCTTGCGACGTTATGCGGT<br>ATTAGCTACCGTTTCCAGT<br>AGTTATCCCCCTCCATCGG<br>GCAGTTTCCCAGACATTAC<br>TCACCCGTCCGCCACTCG<br>TCACCCAAGGAGCAAGCT<br>CCTCTGTGCTACCGTTCG<br>ACTTGCATGTGTTAGGCCT<br>GCCGCCAGCGTTCAATCT<br>GA	**Citrobacter freundii**

Av. Pádua Dias, 11 • Caixa Postal 9 • CEP 13418-900 • Piracicaba, SP • Brasil
Telefone: (19) 3447-6007 ou 3447-6009 – e-mail: labmic@usp.br

Amostra	Sequência F	Sequência R	Nome Científico
T2FE1	ATGCAAGTCGAACGGTA GCACAGAGGAGCTTGC TCCTTGGGTGACGAGT GGCGGACGGGTGAGTA ATGTCTGGGAAACTGCC CGATGGAGGGGGATAA CTACTGGAAACGGTAGC TAATACCGCATAACGTC GCAAGACCAAAGAGGG GGACCTTCGGGCCTCT TGCCATCGGATGTGCC CAGATGGGATTAGCTAG TAGGTGGGGTAACGGC TCACCTAGGCGACGATC CCTAGCTGGTCTGAGA GGATGACCAGCCACAC TGGAACTGAGACACGG TCCAGACTCCTACGGG AGGCAGCAGTGGGGAA TATTGCACAATGGGCGC AAGCCTGATGCAGCCAT GCCGCGTGTATGAAGA AGGCCTTCGGGTTGTAA AGTACTTTCAGCGAGGA GGAAGGTGTTGTGGTTA ATAACCGCAGCAATTGA CGTTACTCGCAGAAGAA GCACCGGCTAACTCCG TGCC	TCTGCGAGTACGTCATCG CTGCGGTTATTAACCACAA CGCCTTCCTCCTCGCTGA AAGTACTTTACAACCCGAA GGCCTTCTTCATACACGC GGCATGGCTGCATCAGGC TTGCGCCCATTGTGCAATA TTCCCCACTGCTGCCTCC CGTAGGAGTCTGGACCGT GTCTCAGTTCCAGTGTGG CTGGTCATCCTCTCAGAC CAGCTAGGGATCGTCGCC TAGGTGAGCCGTTACCCC ACCTACTAGCTAATCCCAT CTGGGCACATCCGATGGC AAGAGGCCCGAAGGTCCC CCTCTTTGGTCTTGCGAC GTTATGGGTATTAGCTACC GTTTCCAGTAGTTATCCCC CTCCATCGGGCAGTTTCC CAGACATTACTCACCCGTC CGCCACTCGTCACCCAAG GAGCAAGCTCCTCTGTGC TACCGTTCGACTTGCATGT GTTAGGCCTGCCGCCAGC GTTCAATC	**Citrobacter freundii**

Av. Pádua Dias, 11 • Caixa Postal 9 • CEP 13418-900 • Piracicaba, SP • Brasil
Telefone: (19) 3447-6007 ou 3447-6009 – e-mail: labmic@usp.br

Amostra	Sequência F	Sequência R	Nome Científico
T2FE2	GCGGACGGGTGAGTAA<br>TGTCTGGGAAACTGCC<br>CGATGGAGGGGGATAA<br>CTACTGGAAACGGTAGC<br>TAATACCGCATAACGTC<br>CCAAGACCAAAGAGGG<br>GGACCTTCGGGCCTCT<br>TGCCCTCGGATGTGCC<br>CAGATGGGATTAGCTAG<br>TAGGTGGGGTAACGGC<br>TCACCTAGGCGACAATC<br>CCTATCTGGTCTGAGAG<br>GATGACCAGCCACACT<br>GGAACTGAGACACGGT<br>CCACACTCCTACGGGA<br>GGCAGCAGTGGGGAAT<br>ATTGCACAATGGGCGCA<br>AGCCTGATGCACCCATG<br>CCGCGTGTATGAAAAAG<br>GCCTTCGGGTTGTAAAG<br>TACTTTCAGCGAGGAG<br>GAAGGCGTTGTGGTTAA<br>TAACCCCGGCGATTGAC<br>GTTACTCGCAGAAAAAA<br>CACCGGCTAACTCCGT<br>GCCAGCAGCCGCGGTA<br>AAAA	CCGAAGGCCTTCTTCATAC<br>ACGCGGCATGGCTGCATC<br>AGGCTTGCGCCCATTGTG<br>CAATATTCCCCACTGCTGC<br>CTCCCGTAGGAGTCTGGA<br>CCGTATCTCAGTTCCAGTG<br>TGGCTGGTCATCCTCTCA<br>GACCAGCTAGGGATCGTC<br>GCCTAGGTGAGCCGTTAC<br>CCCACCTACTAGCTAATCC<br>CATCTGGGCACATCCGAT<br>GGCAAGAGGCCCGAAGG<br>TCCCCCTCTTTGGTCTTGT<br>GACGTTATGCGGTATTAGC<br>TACCGTTTCCAGTAGTTAT<br>CCCCCTCCATCGGGCAGT<br>TTCCCAGACATTACTCACC<br>CGTCCGCCACTCGTCACC<br>CAAGGAGCAAGCTCCTCT<br>GTGCTACCGTTCGACTTG<br>CATGTGTTAGGCCTGCCG<br>CCAGC	**Citrobacter freundii**

ESALQ
USP

DEPARTAMENTO DE
CIÊNCIA E TECNOLOGIA DE ALIMENTOS

LABMIC
Biomol e Micotoxinas

Av. Pádua Dias, 11 • Caixa Postal 9 • CEP 13418-900 • Piracicaba, SP • Brasil
Telefone: (19) 3447-6007 ou 3447-6009 – e-mail: labmic@usp.br

Amostra	Sequência F	Sequência R	Nome Científico
T3FE1	TGCAGGTCGAACGGTA<br>GCACAGAGGAGCTTGC<br>TCCTTGGGTGACGAGT<br>GGCGGACGGGTGAGTA<br>ATGTCTGGGAAACTGCC<br>CGATGGAGGGGGATAA<br>CTACTGGAAACGGTAGC<br>TAATACCGCATAACGTC<br>GCAAGACCAAAGAGGG<br>GGACCTTCGGGCCTCT<br>TGCCATCGGATGTGCC<br>CAGATGGGATTAGCTAG<br>TAGGTGGGGTAACGGC<br>TCACCTAGGCGACGATC<br>CCTAGCTGGTCTGAGA<br>GGATGACCAGCCACAC<br>TGGAACTGAGACACGG<br>TCCAGACTCCTACGGG<br>AGGCAGCAGTGGGGAA<br>TATTGCACAATGGGCGC<br>AAGCCTGATGCAGCCAT<br>GCCGCGTGTATGAAGA<br>AGGCCTTCGGGTTGTAA<br>AGTACTTTCAGCGAGGA<br>GGAAGGCGTTGTGGTT<br>AATAACCGCGGCGATTG<br>ACGTTACTCGCAGAAGA<br>AGCACCGGCTAACTCC<br>GTGCCA	TCTGCGAGTACGTCATCG<br>CTGAGGTTATTAACCACAA<br>CGCCTTCCTCCTCGCTGA<br>AAGTACTTTACAACCCGAA<br>GGCCTTCTTCATACACGC<br>GGCATGGCTGCATCAGGC<br>TTGCGCCCATTGTGCAATA<br>TTCCCCACTGCTGCCTCC<br>CGTAGGAGTCTGGACCGT<br>GTCTCAGTTCCAGTGGGG<br>CTGGTCATCCTCTCAGAC<br>CAGCTAGGGATCGTCGCC<br>TAGGTGAGCCGTTACCCC<br>ACCTACTAGCTAATCCCAT<br>CTGGGCACATCCGATGGC<br>AAGAGGCCCGAAGGTCCC<br>CCTCTTTGGTCTTGCGAC<br>GTTATGCGGTATTAGCTAC<br>CGTTTCCAGTAGTTATCCC<br>CCTCCATCGGGCAGTTTC<br>CCAGACATTACTCACCCGT<br>CCGCCACTCGTCACCCAA<br>GGAGCAAGCTCCTCTGTG<br>CTACCGTTCGACTTGCATG<br>TGTTAGGCCTGCCGCCAG<br>CGTTCAATCTGA	**Citrobacter freundii**

ESALQ
USP

DEPARTAMENTO DE
CIÊNCIA E TECNOLOGIA DE ALIMENTOS

LABMIC
Biomol e Micotoxinas

Av. Pádua Dias, 11 • Caixa Postal 9 • CEP 13418-900 • Piracicaba, SP • Brasil
Telefone: (19) 3447-6007 ou 3447-6009 – e-mail: labmic@usp.br

Amostra	Sequência F	Sequência R	Nome Científico
T1LE1	AAGTCGAACGGTAGCA<br>CAGAGGAGCTTGCTCC<br>TTGGGTGACGAGTGGC<br>GGACGGGTGAGTAATG<br>TCTGGGAAACTGCCCG<br>ATGGAGGGGGATAACTA<br>CTGGAAACGGTAGCTAA<br>TACCGCATAACGTCGCA<br>AGACCAAAGAGGGGGA<br>CCTTCGGGCCTCTTGC<br>CATCGGATGTGCCCAGA<br>TGGGATTAGCTAGTAGG<br>TGGGGTAACGGCTCAC<br>CTAGGCGACGATCCCTA<br>GCTGGTCTGAGAGGAT<br>GACCAGCCACACTGGA<br>ACTGAGACACGGTCCA<br>GACTCCTACGGGAGGC<br>AGCAGTGGGGAATATTG<br>CACAATGGGCGCAAGC<br>CTGATGCAGCCATGCC<br>GCGTGTATGAAGAAGG<br>CCTTCGGGTTGTAAAGT<br>ACTTTCAGCGAGGAGG<br>AAGGYGTTGTGGTTAAT<br>AACCGCAGCGATTGAC<br>GTTACTCGCAGAAGAAG<br>CACCGGCTAACTCCGT<br>GCCA	TTTTGCGAGTACGTCATCG<br>CTGCGGTTATTAACCACAA<br>CGCCTTCCTCCTCGCTGA<br>AAGTACTTTACAACCCGAA<br>GGCCTTCTTCATACACGC<br>GGCATGGCTGCATCAGGC<br>TTGCGCCCATTGTGCAATA<br>TTCCCCACTGCTGCCTCC<br>CGTAGGAGTCTGGACCGT<br>GTCTCAGTTCCAGTGTGG<br>CTGGTCATCCTCTCAGAC<br>CAGCTAGGGATCGTCGCC<br>TAGGTGAGCCGTTACCCC<br>ACCTACTAGCTAATCCCAT<br>CTGGGCACATCCGATGGC<br>AAGAGGCCCGAAGGTCCC<br>CCTCTTTGGTCTTGCGAC<br>GTTATGCGGTATTAGCTAC<br>CGTTTCCAGTAGTTATCCC<br>CCTCCATCGGGCAGTTTC<br>CCAGACATTACTCACCCGT<br>CCGCCACTCGTCACCCAA<br>GGAGCAAGCTCCTCTGTG<br>CTACCGTTCGACTTGCATG<br>TGTTAGGCCTGCCGCCAG<br>CGTTCAATCTG	**Citrobacter freundii**

DEPARTAMENTO DE
CIÊNCIA E TECNOLOGIA DE ALIMENTOS

Av. Pádua Dias, 11 • Caixa Postal 9 • CEP 13418-900 • Piracicaba, SP • Brasil
Telefone: (19) 3447-6007 ou 3447-6009 – e-mail: labmic@usp.br

Amostra	Sequência F	Sequência R	Nome Científico
T2LE2	CATGCAAGTCGAACGGT<br>AGCACAGAGGAGCTTG<br>CTCCTTGGGTGACGAG<br>TGGCGGACGGGTGAGT<br>AATGTCTGGGAAACTGC<br>CCGATGGAGGGGGATA<br>ACTACTGGAAACGGTAG<br>CTAATACCGCATAACGT<br>CGCAAGACCAAAGAGG<br>GGGACCTTCGGGCCTC<br>TTGCCATCGGATGTGCC<br>CAGATGGGATTAGCTAG<br>TAGGTGGGGTAACGGC<br>TCACCTAGGCGACGATC<br>CCTAGCTGGTCTGAGA<br>GGATGACCAGCCACAC<br>TGGAACTGAGACACGG<br>TCCAGACTCCTACGGG<br>AGGCAGCAGTGGGGAA<br>TATTGCACAATGGGCGC<br>AAGCCTGATGCASCCAT<br>GCCGCGTGTATGAAGA<br>AGGCCTTCGGGTTGTAA<br>AGTACTTTCAGCGAGGA<br>GGAAGGCGTTGTGGTT<br>AATAACCGCAGCGATTG<br>ACGTTACTCGCAGAAGA<br>AGCACCGGCTAACTCC<br>GTGCC	ACGCCTTCCTCCTCGCTG<br>AAAGTACTTTACAACCCGA<br>AGGCCTTCTTCATACACGC<br>GGCATGGCTGCATCAGGC<br>TTGCGCCCATTGTGCAATA<br>TTCCCCACTGCTGCCTCC<br>CGTAGGAGTCTGGACCGT<br>GTCTCAGTTCCAGTGTGG<br>CTGGTCATCCTCTCAGAC<br>CAGCTAGGGATCGTCGCC<br>TAGGTGAGCCGTTACCCC<br>ACCTACTAGCTAATCCCAT<br>CTGGGCACATCCGATGGC<br>AAGAGGCCCGAAGGTCCC<br>CCTCTTTGGTCTTGCGAC<br>GTTATGCGGTATTAGCTAC<br>CGTTTCCAGTAGTTATCCC<br>CCTCCATCGGGCAGTTTC<br>CCAGACATTACTCACCCGT<br>CCGCCACTCGTCACCCAA<br>GGAGCAAGCTCCTCTGTG<br>CTACCGTTCGACTTGCATG<br>TGTTAGGCCTGCCGCCAG<br>CGTTCAATCTG	**Citrobacter freundii**

ESALQ USP

DEPARTAMENTO DE
CIÊNCIA E TECNOLOGIA DE ALIMENTOS

Av. Pádua Dias, 11 • Caixa Postal 9 • CEP 13418-900 • Piracicaba, SP • Brasil
Telefone: (19) 3447-6007 ou 3447-6009 – e-mail: labmic@usp.br

LABMIC Biomol e Micotoxinas

Amostra	Sequência F	Sequência R	Nome Científico
T2LE3	GCAAGTCGAACGGTAG<br>CACAGAGGAGCTTGCT<br>CCTTGGGTGACGAGTG<br>GCGGACGGGTGAGTAA<br>TGTCTGGGAAACTGCC<br>CGATGGAGGGGGATAA<br>CTACTGGAAACGGTAGC<br>TAATACCGCATAACGTC<br>GCAAGACCAAAGAGGG<br>GGACCTTCGGGCCTCT<br>TGCCATCGGATGTGCC<br>CAGATGGGATTAGCTAG<br>TAGGTGGGGTAACGGC<br>TCACCTAGGCGACGATC<br>CCTAGCTGGTCTGAGA<br>GGATGACCAGCCACAC<br>TGGAACTGAGACACGG<br>TCCAGACTCCTACGGG<br>AGGCAGCAGTGGGGAA<br>TATTGCACAATGGGCGC<br>AAGCCTGATGCAGCCAT<br>GCCGCGTGTATGAAGA<br>AGGCCTTCGGGTTGTAA<br>AGTACTTTCAGCGAGGA<br>GGAAGGCGTTGTGGTT<br>AATAACCGCGGCGATTG<br>ACGTTACTCGCAGAAGA<br>AGCACCGGCTAACTCC<br>GTGCC	CGAGTACGTCATCGCTGC<br>GGTTATTAACCACAACGCC<br>TTCCTCCTCGCTGAAAGTA<br>CTTTACAACCCGAAGGCC<br>TTCTTCATACACGCGGCAT<br>GGCTGCATCAGGCTTGCG<br>CCCATTGTGCAATATTCCC<br>CACTGCTGCCTCCCGTAG<br>GAGTCTGGACCGTGTCTC<br>AGTTCCAGTGTGGCTGGT<br>CATCCTCTCAGACCAGCTA<br>GGGATCGTCGCCTAGGTG<br>AGCCGTTACCCCACCTACT<br>AGCTAATCCCATCTGGGC<br>ACATCCGATGGCAAGAGG<br>CCCGAAGGTCCCCCTCTT<br>TGGTCTTGCGACGTTATG<br>CGGTATTAGCTACCGTTTC<br>CAGTAGTTATCCCCCTCCA<br>TCGGGCAGTTTCCCAGAC<br>ATTACTCACCCGTCCGCC<br>ACTCGTCACCCAAGGAGC<br>AAGCTCCTCTGTGCTACC<br>GTTCGACTTGCATGTGTTA<br>GGCCTGCCGCCAGCGTTC<br>AATC	**Citrobacter freundii**

DEPARTAMENTO DE
CIÊNCIA E TECNOLOGIA DE ALIMENTOS
LABMIC Biomol e Micotoxinas
Av. Pádua Dias, 11 • Caixa Postal 9 • CEP 13418-900 • Piracicaba, SP • Brasil
Telefone: (19) 3447-6007 ou 3447-6009 – e-mail: labmic@usp.br

Amostra	Sequência F	Sequência R	Nome Científico
T3LE1	GTCGAGCGGTAGCACA<br>GAGGACTTTGTCTCTTG<br>TGTGACGAGTGGGGCA<br>GGGGTGTGAAGTGTGT<br>GGGAAACTGTGCTATG<br>GGGGGGGATATCTTCTG<br>GAAACGGGATCTTATAC<br>CGCATAATCTCCCGACA<br>CCAATGAGGGGGACCC<br>TCGCGCCTCTTGCCCT<br>CATGTGTCCCCATAGGG<br>GATTATCTAATGTGGGG<br>GGTAACGTCTCCCCTAC<br>GAGAATATCTCTCTGGG<br>GTGTGAGAAGAAGACC<br>ACCCACTCTGGATCAGA<br>GACACGCCCCACACTC<br>CCACGGGGGGCAGCAG<br>TGGGGAATATTGCACAG<br>TGGGCGCGACTCTGAT<br>ACACCCGTCCCGCGTG<br>TGTGAAAAACGTCTTCG<br>TTGTGTAAAACTCTTTC<br>CGCGAAGAAAAGCGAG<br>GTGGGTATAAACCCCGC<br>CGCTAGTGACTTCCCTC<br>ACAAAAAACACCCCCTA<br>TCTCTCTGCG	GGATATTAACCACAACAGC<br>CTTCCTCCTCGCTGAAAG<br>TAGCTTTACAACCCGAAG<br>GCCTTCTTCATACACGCG<br>GCATGGCTGCATCAGGCT<br>TGCGCCCATTGTGCAATAT<br>TCCCCACTGCTGCCTCCC<br>GTAGGAGTCTGGACCGTG<br>TCTCAGTTCCAGTGTGGC<br>TGATCATCCTCTCAGACCA<br>GCTAGGGATCGTCGCCTA<br>GGTGAGCCGTTACCCCAC<br>CTACTAGCTAATCCCATCT<br>GGGCACATCCGATGGCAA<br>GAGGCCCGAAGGTCCCC<br>CTCTTTGGTCTTGCGACG<br>TTATGCGGTATTAGCTACC<br>GTTTCCAGTAGTTATCCCC<br>CTCCATCGGGCAGTTTCC<br>CAGACATTACTCACCCGTC<br>CGCCGCTCGTCACCCAAG<br>GAGAAAGCCCCTCTGCGC<br>CCCCGCGCCACTTGTGTG<br>TGTGAGGTGTGCCCCCCG<br>CGCTCTCTCTGAGACGG	Não identificada

DEPARTAMENTO DE
CIÊNCIA E TECNOLOGIA DE ALIMENTOS
Av. Pádua Dias, 11 • Caixa Postal 9 • CEP 13418-900 • Piracicaba, SP • Brasil
Telefone: (19) 3447-6007 ou 3447-6009 – e-mail: labmic@usp.br

Amostra	Sequência F	Sequência R	Nome Científico
T3HV1	CCTACACATGCAAGTCG<br>AGCGGCAGCGGGAAAG<br>TAGCTTGCTACTTTTGC<br>CGGCGAGCGGCGGAC<br>GGGTGAGTAATGCCTG<br>GGAAATTGCCCAGTCG<br>AGGGGGATAACAGTTG<br>GAAACGACTGCTAATAC<br>CGCATACGCCCTACGG<br>GGGAAAGCAGGGGACC<br>TTCGGGCCTTGCGCGA<br>TTGGATATGCCCAGGGG<br>GGATTAGCTAGTTGGTG<br>GGGTAATGGCTCACCAA<br>GGGGACGATCCCTAGC<br>TGGTCTGAGAGGATGAT<br>CAGCCACACTGGAACT<br>GAGACACGGTCCAGAC<br>TCCTACGGGAGGCAGC<br>AGTGGGGAATATTGCAC<br>AATGGGGGAAACCCTG<br>RTGCAGCCATGCCGCG<br>TGTGTGAARAAGGCCTT<br>CGGGTTGTAAAGCACTT<br>TCAGCGAGGAGGAAAG<br>GTCGGTGGCTAATATCC<br>GACGGCTGTGACGTTA<br>CTCGCAGAAGAAGCAC<br>CGGCTAACTC	CCTTTCCTCCTCGCTGAA<br>AGTGCTTTACAACCCGAA<br>GGCCTTCTTCACACACGC<br>GGCATGGCTGCATCAGGG<br>TTTCCCCCATTGTGCAATA<br>TTCCCCACTGCTGCCTCC<br>CGTAGGAGTCTGGACCGT<br>GTCTCAGTTCCAGTGTGG<br>CTGATCATCCTCTCAGACC<br>AGCTAGGGATCGTCGCCT<br>TGGTGAGCCATTACCCCA<br>CCAACTAGCTAATCCCACC<br>TGGGCATATCCAATCGCG<br>CAAGGCCCGAAGGTCCCC<br>TGCTTTCCCCCGTAGGGC<br>GTATGCGGTATTAGCAGTC<br>GTTTCCAACTGTTATCCCC<br>CTCGACTGGGCAATTTCC<br>CAGGCATTACTCACCCGT<br>CCGCCGCTCGCCGGCAA<br>AAGTAGCAAGCTACTTCCC<br>CGCTGCCGCTCGACTTGC<br>ATGTGTTAGGCCTGCCGC<br>CAGCGTTCAATCT	Aeromonas sp

Av. Pádua Dias, 11 • Caixa Postal 9 • CEP 13418-900 • Piracicaba, SP • Brasil
Telefone: (19) 3447-6007 ou 3447-6009 – e-mail: labmic@usp.br

Amostra	Sequência F	Sequência R	Nome Científico
T3FV1	TGCAGTCGAGCGGCAGCGGGGA AGTAGCTTGCTACTTTTGCCGGC GAGCGGCGGACGGGTGAGTAAT GCCTGGGAAATTGCCCAGTCGA GGGGGATAACAGTTGGAAACSAC TGCTAATACCGCATACGCCCTACG GGGGAAAGCAGGGGACCTTCGG GCCTTGCGCGATTGGATATGCCC AGGTGGGATTAGCTAGTTGGTGG GGTAATGGCTCACCAAGGSGACG ATCCCTAGCTGGTCTGAGAGGAT GATCAGCCACACTGGAACTGAGA CACGGTCCAGACTCCTACGGGA GGCAGCAGTGGGGAATATTGCAC AATGGGGGAAACCCTGATGCAGC CATGCCGCGTGTGTGAAGAAGG CCTTCGGGTTGTAAAGCACTTTC AGCGAGGAGGAAAGG	CTTTCCTCCTCGCTGAAAGTGC TTTACAACCCGAAGGCCTTCTT CACACACGCGGCATGGCTGCAT CAGGGTTTCCCCCATTGTGCAA TATTCCCCACTGCTGCCTCCCG TAGGAGTCTGGACCGTGTCTCA GTTCCAGTGTGGCTGATCATCC TCTCAGACCAGCTAGGGATCGT CGCCTTGGTGAGCCATTACCCC ACCAACTAGCTAATCCCACCTG GGCATATCCAATCGCGCAAGGC CCGAAGGTCCCCTGCTTTCCCC CGTAGGGCGTATGCGGTATTAG CAGTCGTTTCCAACTGTTATCCC CCTCGACTGGGCAATTTCCCAG GCATTACTCACCCGTCCGCCGC TCGCCGGCAAAAGTAGCAAGCT ACTTCCCCGCTGCCGCTCGACT TGCATGTGTTAGGCCTGCCGC	Aeromonas sp

ESALQ USP

DEPARTAMENTO DE
CIÊNCIA E TECNOLOGIA DE ALIMENTOS

LABMIC Biomol e Micotoxinas

Av. Pádua Dias, 11 • Caixa Postal 9 • CEP 13418-900 • Piracicaba, SP • Brasil
Telefone: (19) 3447-6007 ou 3447-6009 – e-mail: labmic@usp.br

Amostra	Sequência F	Sequência R	Nome Científico
T2FE3	TACACATGCAGTCGAACGGTAAC<br>ASGAAGSAGCTTGCTSCTTCGGT<br>GACGAGTGGCGGACGGGTGAGT<br>AATGTCTGGGAAACTGCCCGATG<br>GAGGGGGATAACTACTGGAAACG<br>GTAGCTAATACCGCATAACGTCGC<br>AAGACCAAAGAGGGGGACCCTC<br>GGGCCTCTTGCCATCGGATGTGC<br>CCAGATGGGATTAGCTAGTAGGT<br>GGGGTAACGGCTCACCTAGGCG<br>ACGATCCCTAGCTGGTCTGAGAG<br>GATGACCAGCCACACTGGAACTG<br>AGACACGGTCCAGACTCCTACGG<br>GAGGCAGCAGTGGGGAATATTGC<br>ACAATGGGCGCAAGCCTGATGCA<br>GCCATGCCGCGTGTATGAAGAAG<br>GCCTTCGGGTTGTAAAGTACTTT<br>CAGCGGGGAGGAAGGCGTTGAG<br>GTTAATAACCTTGTCGATTGACGT<br>TACCCGCAGAAGAAGCACCGGCT<br>AACTCCGTGC	CAGTCGAACGGTAACAGGAAGG<br>AGCTTGCTGCTTCGGTGACGAG<br>TGGCGGACGGGTGAGTAATGTC<br>TGGGAAACTGCCCGATGGAGG<br>GGGATAACTACTGGAAACGGTA<br>GCTAATACCGCATAACGTCGCAA<br>GACCAAAGAGGGGGACCCTCG<br>GGCCTCTTGCCATCGGATGTGC<br>CCAGATGGGATTAGCTAGTAGG<br>TGGGGTAACGGCTCACCTAGGC<br>GACGATCCCTAGCTGGTCTGAG<br>AGGATGACCAGCCACACTGGAA<br>CTGAGACACGGTCCAGACTCCT<br>ACGGGAGGCAGCAGTGGGGAA<br>TATTGCACAATGGGCGCAAGCC<br>TGATGCAGCCATGCCGCGTGTA<br>TGAAGAAGGCCTTCGGGTTGTA<br>AAGTACTTTCAGCGGGGAGGAA<br>GGCGTTGAGGTTAATAACCTTGT<br>CGATTGACGTTACCCGCAGAAG<br>AAGCACCGGCTAACT	**Enterobacter hormaechei**

DEPARTAMENTO DE
CIÊNCIA E TECNOLOGIA DE ALIMENTOS
Av. Pádua Dias, 11 • Caixa Postal 9 • CEP 13418-900 • Piracicaba, SP • Brasil
Telefone: (19) 3447-6007 ou 3447-6009 – e-mail: labmic@usp.br

Amostra	Sequência F	Sequência R	Nome Científico
T3LV1	GCTACACATGCAAGTCGAGCGGC<br>AGCGGGAAAGTAGCTTGCTACTT<br>TTGCCGGCGAGCGGCGGACGGG<br>TGAGTAATGCCTGGGGATCTGCC<br>CARTCGAGGGGGATAACTACTGG<br>AAACGGTAGCTAATACCGCATACG<br>CCCTACGGGGGAAAGCAGGGGA<br>CCTTCGGGCCTTGCGCGATTGGA<br>TGAACCCAGGTGGGATTAGCTAG<br>TTGGTGAGGTAATGGCTCACCAA<br>GGCGACGATCCCTAGCTGGTCTG<br>AGAGGATGATCAGCCACACTGGA<br>ACTGAGACACGGTCCAGACTCCT<br>ACGGGAGGCAGCAGTGGGGAAT<br>ATTGCACAATGGGGGAAACCCTG<br>ATGCAGCCATGCCGCGTGTGTGA<br>AGAAGGCCTTCGGGTTGTAAAGC<br>ACTTTCAGCGAGGAGGAAAGGTT<br>GGTAGCTAATAACTGCCAGCTGT<br>GACGTTACTCGCAGAAGAAGCAC<br>CGGCTAACTCCGTGGCGGCAGC<br>CGCGGTAAA	TCTGCGAGTACGTCACAGCTGG<br>CAGTTATTAGCTACCAACCTTTC<br>CTCCTCGCTGAAAGTGCTTTAC<br>AACCCGAAGGCCTTCTTCACAC<br>ACGCGGCATGGCTGCATCAGG<br>GTTTCCCCCATTGTGCAATATTC<br>CCCACTGCTGCCTCCCGTAGGA<br>GTCTGGACCGTGTCTCAGTTCC<br>AGTGTGGCTGATCATCCTCTCA<br>GACCAGCTAGGGATCGTCGCCT<br>TGGTGAGCCATTACCTCACCAA<br>CTAGCTAATCCCACCTGGGTTCA<br>TCCAATCGCGCAAGGCCCGAAG<br>GTCCCCTGCTTTCCCCCGTAGG<br>GCGTATGCGGTATTAGCTACCGT<br>TTCCAGTAGTTATCCCCCTCGAC<br>TGGGCAGATCCCCAGGCATTAC<br>TCACCCGTCCGCCGCTCGCCG<br>GCAAAAGTAGCAAGCTACTTTC<br>CCGCTGCCGCTCGACTTGCATG<br>TGTTAGGCCTGCCGCCAGCGTT<br>CAATCTGAGCAAGGATCAAACT<br>CA	**Aeromonas sp.**

DEPARTAMENTO DE
CIÊNCIA E TECNOLOGIA DE ALIMENTOS

ESALQ USP

LABMIC Biomol e Micotoxinas

Av. Pádua Dias, 11 • Caixa Postal 9 • CEP 13418-900 • Piracicaba, SP • Brasil
Telefone: (19) 3447-6007 ou 3447-6009 – e-mail: labmic@usp.br

Amostra	Sequência F	Sequência R	Nome Científico
T3LE2	GTCGAGCGGCAGCGGGAAAGTA<br>GCTTGCTACTTTTGCCGGCGAGC<br>GGCGGACGGGTGAGTAATGCCT<br>GGGGATCTGCCCAGTCGAGGGG<br>GATAACTACTGGAAACGGTAGCT<br>AATACCGCATACGCCCTACGGGG<br>GAAAGCAGGGGACCTTCGGGCC<br>TTGCGCGATTGGATGAACCCAGG<br>TGGGATTAGCTAGTTGGTGAGGT<br>AATGGCTCACCAAGGCGACGATC<br>CCTAGCTGGTCTGAGAGGATGAT<br>CAGCCACACTGGAACTGAGACAC<br>GGTCCAGACTCCTACGGGAGGC<br>AGCAGTGGGGAATATTGCACAAT<br>GGGGGAAACCCTGATGCAGCCA<br>TGCCGCGTGTGTGAAGAAGGCC<br>TTCGGGTTGTAAAGCACTTTCAG<br>CGAGGAGGAAAGGTTGGTAGCTA<br>ATAACTGCCAGCTGTGACGTTAC<br>TCGCAAAAGAAGCACCGGCTAAC<br>TCCGTGCCAC	TACGTCACTGTGCTGCAGTTATT<br>AGCTACACACCCTTTCCTCCTC<br>GCTGAAAGTAGCTTTACAACCC<br>GAAGGCCTTCTTCACACACGCG<br>GCATGGCTGCATCAGGGTTTCC<br>CCCATTGTGCAATATTCCCCACT<br>GCTGCCTCCCGTAGGAGTCTGG<br>ACCGTGTCTCAGTTCCAGTGTG<br>GCTGATCATCCTCTCAGACCAG<br>CTAGGGATCGTCGCCTTGGTGA<br>GCCATTACCTCACCAACTAGCTA<br>ATCCCACCTGGGTTCATCCAATC<br>GCGCAAGGCCCGAAGGTCCCC<br>TGCTTTCCCCCGTAGGGCGTAT<br>GCGGTATTAGCTACCGTTTCCA<br>GTAGTTATCCCCCTCGACTGGG<br>CAGATCCCCAGGCATTACTCAC<br>CCGTCCGCCGCTCGCCGGCAA<br>AAGTAGCAAGCTACTTTCCCGC<br>TGCCGCTCGACTTGCATGTGTT<br>AGGCCTGCCGCCAGCGTTCAAT<br>CTGAGCCTGA	**Aeromonas veronii**

Ficamos à disposição para maiores esclarecimentos.

Piracicaba, 04 de novembro de 2025.

Alsmellcesar

Prof[a]. Dr[a]. Aline Silva Mello Cesar
ESALQ/USP
