# Supplementary material for: Detection of Bacterial Internalization in Lettuce (Lactuca sativa) Leaves Grown in Aquaponic Systems with Nile Tilapia (Oreochromis niloticus) Under Microbial Challenge
Source: Biology (Basel). 2026 Mar 31;15(7):559. doi: 10.3390/biology15070559 (PMC13072089; doi:10.3390/biology15070559)
Supplement: Supplementary file 1 [file biology-15-00559-s001.zip › File S3 File Ethical approval Certificate.pdf]

CERTIFICADO

Certificamos que a proposta intitulada "Avaliação da contaminação microbiológica de vegetais cultivados em sistema de aquaponia com tilápia-do-Nilo (Oreochromis niloticus) infectada por bactérias patogênicas", protocolada sob o CEUA nº 1853260225 (ID 000612), sob a responsabilidade de **Brunno da Silva Cerozi** *e equipe; Angélica Adiação Jossefa* - que envolve a produção, manutenção e/ou utilização de animais pertencentes ao filo Chordata, subfilo Vertebrata (exceto o homem), para fins de pesquisa científica ou ensino - está de acordo com os preceitos da Lei 11.794 de 8 de outubro de 2008, com o Decreto 6.899 de 15 de julho de 2009, bem como com as normas editadas pelo Conselho Nacional de Controle da Experimentação Animal (CONCEA), e foi **APROVADA** pela Comissão de Ética no Uso de Animais da Escola Superior de Agricultura (CEUA/ESALQ) na reunião de 17/03/2025.

We certify that the proposal "Evaluation of microbiological contamination of veget.a.b.l.e.s grown in aquaponic system with Nile tilapia (Oreochromis niloticus) infected by pathogenic bacteria", utilizing 135 Fishes (135 males), protocol number CEUA 1853260225 (ID 000612), under the responsibility of **Brunno da Silva Cerozi** *and team; Angélica Adiação Jossefa* - which involves the production, maintenance and/or use of animals belonging to the phylum Chordata, subphylum Vertebrata (except human beings), for scientific research purposes or teaching - is in accordance with Law 11.794 of October 8, 2008, Decree 6899 of July 15, 2009, as well as with the rules issued by the National Council for Control of Animal Experimentation (CONCEA), and was **APPROVED** by the Ethic Committee on Animal Use of the Luiz de Queiroz College of Agriculture - ESALQ (CEUA/ESALQ) in the meeting of 03/17/2025.

UNIVERSIDADE DE SÃO PAULO
Escola Superior de Agricultura "Luiz de Queiroz"
SVAPESQ-11
ESALQ

Finalidade da Proposta: Pesquisa (Acadêmica)

Vigência da Proposta: de 04/2025 a 08/2025 Área: Ciência Animal E Pastagens

Origem: Campus Luiz de Queiroz (ESALQ)
Espécie: Peixes sexo: Machos idade: 2 a 3 meses Quantidade: 135
Linhagem: Nilótica Peso: 10 a 20 g

Piracicaba, 17 de março de 2025

Prof. Dr. Roberto Sartori Filho
Coordenador da Comissão de Ética no Uso de Animais
Escola Superior de Agricultura

Prof. Dr. Gerson Barreto Mourão
Vice-Coordenador da Comissão de Ética no Uso de Animais
Escola Superior de Agricultura
