# Supplementary figures and images for: Detection of Bacterial Internalization in Lettuce (Lactuca sativa) Leaves Grown in Aquaponic Systems with Nile Tilapia (Oreochromis niloticus) Under Microbial Challenge
Source: Biology (Basel). 2026 Mar 31;15(7):559. doi: 10.3390/biology15070559 (PMC13072089; doi:10.3390/biology15070559)

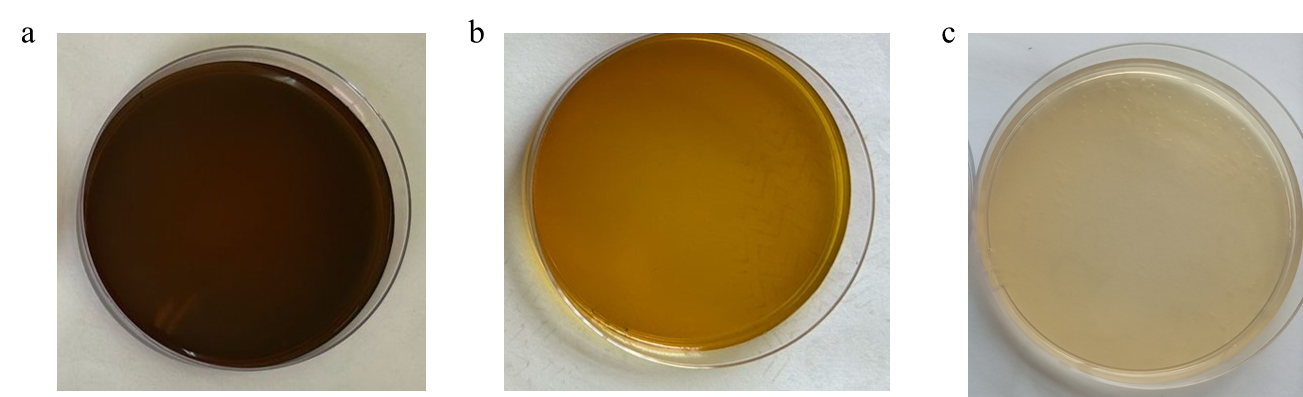

Supplement: Supplementary file 1 [file biology-15-00559-s001.zip › Figure S1 Results of microbiological analysis of samples from disinfection processes. (a) EMB for E. coli (b) TCBS for V. cholerae (c) TAS—non-selective agar.tiff]

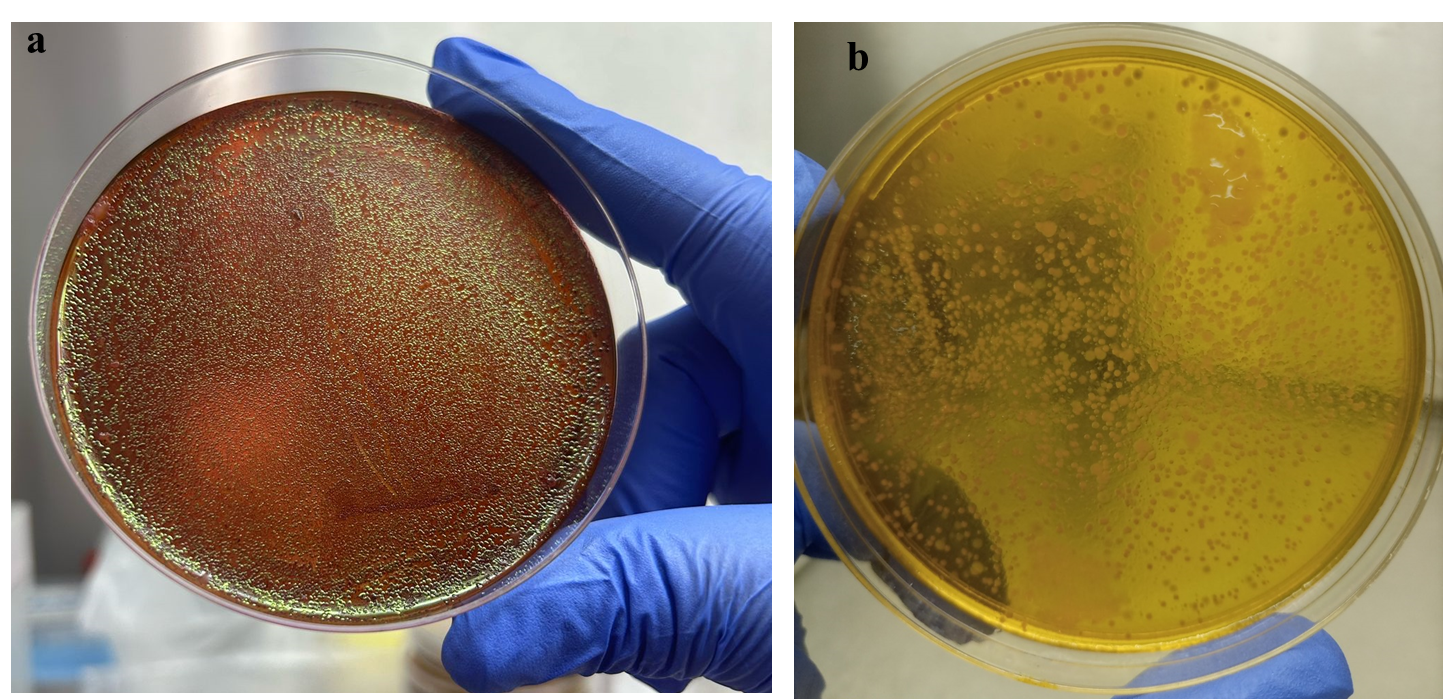

Supplement: Supplementary file 1 [file biology-15-00559-s001.zip › Figure S2 Presumptive colonies results. (a) EMB for E. coli and (b) TCBS for V. cholerae.tiff]

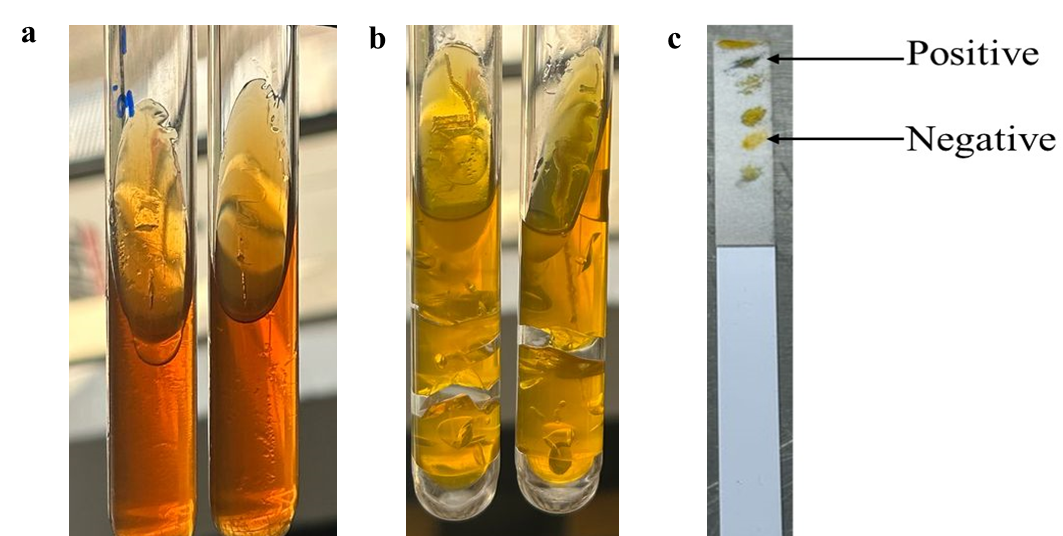

Supplement: Supplementary file 1 [file biology-15-00559-s001.zip › Figure S3 Results of biochemistry tests. (a) negative for E. coli in TSI agar, (b) positive for E. coli in TSI, and (c) positive and negative for V. cholerae in oxidase test..tiff]
